# Biosensors to Monitor Cell Activity in 3D Hydrogel-Based Tissue Models

**DOI:** 10.3390/s22041517

**Published:** 2022-02-15

**Authors:** Arianna Fedi, Chiara Vitale, Paolo Giannoni, Guido Caluori, Alessandra Marrella

**Affiliations:** 1 National Research Council of Italy, Institute of Electronics, Computer and Telecommunication Engineering (IEIIT), 16149 Genoa, Italy; arianna.fedi@ieiit.cnr.it (A.F.); chiara.vitale@ieiit.cnr.it (C.V.); 2 Department of Computer Science, Bioengineering, Robotics and Systems Engineering (DIBRIS), University of Genoa, 16126 Genoa, Italy; 3 Department of Experimental Medicine (DIMES), University of Genoa, 16132 Genoa, Italy; paolo.giannoni@unige.it; 4 IHU LIRYC, Electrophysiology and Heart Modeling Institute, Fondation Bordeaux Université, 33600 Pessac, France; guido.caluori@ihu-liryc.fr; 5 INSERM UMR 1045, Cardiothoracic Research Center of Bordeaux, University of Bordeaux, 33600 Pessac, France

**Keywords:** biosensors, hydrogels, 3D models, in vitro cell cultures

## Abstract

Three-dimensional (3D) culture models have gained relevant interest in tissue engineering and drug discovery owing to their suitability to reproduce in vitro some key aspects of human tissues and to provide predictive information for in vivo tests. In this context, the use of hydrogels as artificial extracellular matrices is of paramount relevance, since they allow closer recapitulation of (patho)physiological features of human tissues. However, most of the analyses aimed at characterizing these models are based on time-consuming and endpoint assays, which can provide only static and limited data on cellular behavior. On the other hand, biosensing systems could be adopted to measure on-line cellular activity, as currently performed in bi-dimensional, i.e., monolayer, cell culture systems; however, their translation and integration within 3D hydrogel-based systems is not straight forward, due to the geometry and materials properties of these advanced cell culturing approaches. Therefore, researchers have adopted different strategies, through the development of biochemical, electrochemical and optical sensors, but challenges still remain in employing these devices. In this review, after examining recent advances in adapting existing biosensors from traditional cell monolayers to polymeric 3D cells cultures, we will focus on novel designs and outcomes of a range of biosensors specifically developed to provide real-time analysis of hydrogel-based cultures.

## 1. Introduction

### 1.1. Standard 2D Cultures and In Vivo Models

Two-dimensional (2D) cells monolayers, cultured over planar substrates, have been considered the main in vitro culture systems for cell-based screening and drug testing for several decades [1,2]. These systems offer simple, cheap and relatively standardized tools for reproducing biological processes and study (patho)physiological mechanisms in response to changes in the intra/extracellular environment since the late 19th century [3,4]. However, these bi-dimensional platforms display some significant disadvantages, principally due to the lack of a three-dimensional (3D) tissue-specific spatial features, as reported in Table 1. Planar models present thus incomplete or altered cell-to-cell and cell-to-matrix interactions, as well as tissue-specific biomechanical and biochemical cues; this, in turn, may affect normal cells proliferation and differentiation, genes and proteins expression, and response to pharmacological treatments [5,6]. Conventional 2D culture on plastic may change the original cell morphology and heterogeneity [7,8], as cells are mostly in contact with surface coating proteins through focal adhesions, forcing a polarized and an abnormally flattened shape, with fewer contacts available for intercellular connection [9,10]. The spatial architecture of cell communication is, in fact, pivotal for correct cell functions and cannot be reproduced correctly in a bi-dimensional context, where cells cannot uniformly express adhesion molecules and receptors [11] and are completely exposed to nutrients and oxygen distributions, in contrast to the metabolic gradients experienced in vivo [12,13,14,15]. Similarly, although some drugs appear to be efficient in vitro by using 2D systems, they might not be as effective when administered in vivo, often providing false positive results [16,17,18].

On the other hand, animal models, which are considered the current gold standard, present other disadvantages, such as being time-consuming, remarkably laborious, expensive [19], and not fully translatable to human scenarios [20]. Moreover, animal testing has been subjected to ethical issues, objections, and limitative regulations as testified by the ban of animal models for cosmetic testing recently promoted in EU countries [21,22,23,24,25]. Furthermore, the establishment of 3Rs-based approaches aims at sensitizing scientists to optimize their animal employment and slowly promoting alternative in vitro models [26,27].

### 1.2. 3D Tissue Models

In this context, in vitro 3D cell culture models have consequently been gaining attention as compromises between 2D cultures and animal models [16,28]. In fact, 3D systems can better reproduce the in vivo environment, if compared with 2D cultures, while maintaining the benefits of the traditional bi-dimensional cultures—high control of the experimental conditions, ease of manipulation [1,29,30,31,32]. 

In this scenario, different typologies of 3D in vitro models can be identified. One of them consists in the cellular spheroid, which is a spontaneous and stable cell aggregate [32,33]. It has been shown that it recapitulates different biological features (e.g., 3D architecture, chemical gradients, hypoxic core) better than cell monolayers [34]. In particular, cells display interactions with the neighbors in all spatial directions and exhibit a proliferation rate comparable to that observed in vivo, often significantly lower than the one shown in 2D culture conditions [35,36]. Although these 3D culture systems widely improved the reliability of in vitro tests in different medical areas, they still have different drawbacks, mainly caused by a general lack of a proper surrounding extracellular matrix (ECM). In fact, the tissue microenvironmental conditions, such as the matrix mechanical rigidity and its role in modulating cell activity, cannot be finely tuned and reproduced [37]. 

To provide cells of the necessary ECM, researchers moved to the culture of 3D engineered tissues [38,39,40]. For this purpose, different kinds of biomaterials have been adopted [40,41,42].

#### 3D Hydrogel-Based Tissue Models

3D hydrogel-based tissue models are emerging among others due to their potential of mimicking native ECM and their cell encapsulation capability [41].

In particular, hydrogels are hydrophilic polymer chains embedded in water-based 3D substrates, where cells can be entrapped to resemble, in vitro, specific tissue complexities [42], and where molecules can diffuse through their reticulated structure, as it happens in human organs [43,44,45,46]. They can more closely recapitulate key mechanical and biochemical features of the human tissues due to their high water content, softness, and reticulated structure. In particular, these soft polymeric substrates better replicate most of the soft tissues in the human body [47,48], allowing proper oxygen, nutrients and signaling molecules transport, as well as cells migration and arrangement [49,50,51]. 

Actually, a plethora of various types of hydrogels with different physical, chemical, biological features have been explored [52]. All of them need to be carefully optimized based on the cell types used and the final tissue to resemble. In fact, besides a proper level of porosity mentioned above to guarantee an efficient transport of nutrients, they should also present adequate mechanical properties to promote cell attachment, arrangement and tissue formation [53,54]. Natural polymeric biomaterials such as collagen, gelatin and hyaluronic acid are the most used to fabricate 3D hydrogels because of their chemical resemblance of the natural ECM components [55].

### 1.3. Importance of Integrating Biosensors with 3D Hydrogel-Based Tissue Models 

When culturing a 3D hydrogel-based tissue model, cells morphology, viability and proliferation are generally assessed by using optical/confocal microscopes, but, unfortunately, optical microscopy is hampered in the visualization of structural details in such 3D constructs [56]. In this case, confocal fluorescence microscopy allows a more precise visualization of the 3D structure [57], but data acquisition on a large enough number of replicas to perform statistically robust quantitative analysis is very time-consuming. Moreover, it is often of interest to monitor the actual state of the cells and the continuous observation of morphological parameters can only contribute to provide elemental information about the cellular behavior. Furthermore, the use of fluorescent staining, as an alternative—e.g., in live/dead assays—is laborious and involves irreversible cellular modifications and damages, providing only end-point information [17,58]. 

Hence, the need for more sophisticated non-disruptive systems to monitor the cellular physiological state in real-time, over time and upon different stimulations, is perceived as primary for the development standardized advanced culture models.

In this context, the use of biosensors can be of relevant importance to acquire the actual state of the cells, as well as to collect sufficient data for robust statistics in a non-invasive way. 

### 1.4. Biosensors 

The definition of biosensor is “a self-contained analytical device that combines a biological component with a physicochemical component for the detection of an analyte of biological importance” [59]. 

Generally, a biosensor is composed of a receptor of biological sample, a transducer and a system to detect the results and convert them into a measurable signal [60]. Thanks to their components, biosensors can measure very small signals from few biological samples, providing a robust analysis in a non-invasive way [61]. 

They are designed to detect the presence of an analyte as an indirect measure of several cellular phenomena. Among various principles to classify biosensors, they can be categorized based on the physico-chemical parameter monitored as a measure of the cellular activity. 

For example, the main molecules involved in cell metabolism (pH, oxygen, glucose, etc.) can be analyzed through specific biosensors [62]. The integration of advanced in vitro models with an accurate metabolic analysis is pushed by different needs. In studies related to drug efficacy tests, the monitoring of metabolism contributes to the standardization of the tests, and, especially in cancer research, provides important information on tumor progression and the eventual efficacy of anticancer drugs [63]. Different working principles are adopted in designing this category of biosensors. In general, electrochemical and optical-based ones are the most commonly used, which enable a label-free, continuous monitoring of metabolic transient mechanisms [64].

Information related to cellular activity can be provided also by detecting the variations on the cellular electrical properties. In this case, impedance-based biosensors, which reveal changes in conductivity as indirect measure of cell growth, are the most commonly used for label-free detection, since this method does not require any detection tag for sensing of the analytes [61]. 

Another important category of biosensors is represented by the molecule-based ones, which exploit biochemical reactions to detect the presence of specific molecules secreted by the cells [65]. In particular, within these systems a bio-recognition element, such as an antibody or an aptamer, is immobilized close to the cells. The combination of high-affinity biomolecules with cells allows a higher level of sensitivity and selectivity of a range of analytes, if compared to other categories of biosensors [66].

Overall, there has been a huge progress both in the development of 3D hydrogel-based tissue models and biosensors technologies, but their integrated adoption into standardized cell culture platforms is still in its infancy.

Several kinds of biosensors, based on different working principles, have already been realized and validated to work in bi-dimensional environments and applied in different fields, due to their attractive features, such as stability and sensitivity, ease of miniaturization and cheapness, which make them suitable for many different biomedical applications [67,68].

Henceforward on, in this review, we will examine in detail: (i) the current strategies to adapt different kinds of biosensors, originally conceived for 2D settings, to several 3D cell hydrogel-based culture models, by taking advantage of the same working principles adopted in 2D cell cultures; and (ii) the cutting-edge technologies to realize brand-new application-specific biosensors for monitoring the activity of cells cultured in 3D advanced hydrogel-based tissue models. 

This critical review is divided in different sections based on the physico-chemical parameters monitored as indirect measures of the status of the cells:

- biosensors to monitor the cellular metabolism, where glucose and oxygen consumptions as well as pH surrounding levels are monitored;
- impedance biosensors, where the variations of cellular electrical properties are revealed;
- biosensors to detect the secretion of specific molecules modulated by cell activity.

## 2. Biosensors to Monitor the Cellular Metabolism

Cellular metabolism represents the complex biological mechanism, through which living cells uptake energetic substrates (e.g., glucose, fatty acids, etc.) and oxygen (O_2_), to generate energy and acidic waste products [69,70]. Cells release acidic metabolites into the surrounding microenvironment (causing extracellular acidification) to maintain the proper intracellular pH levels [71]. The continuous monitoring of these parameters provides information on the status of the cells. In order to analyze metabolism, the most relevant metabolic parameters, e.g., pH, oxygen or glucose, are generally measured, through standard end-point assays [63]. We here discuss different biosensors, for pH, O_2_ or glucose monitoring, integrated with 3D hydrogel-based culture formats.

### 2.1. PH Biosensors

pH level in the microenvironment regulates several pivotal cellular functions [72,73]; an altered pH, for example, is an emerging hallmark of cancer advancement [74]. An acidic pH (6.2–6.9), with respect to the neutral extracellular pH of normal cells (7.2–7.4), enables, for example, disease progression by promoting cancer cells proliferation, migration and invasion [75,76,77]. Accordingly, the measurement of pH levels is crucial to monitor cells conditions, revealing changes related to the cell status and phase. 

Three main features should be considered in designing sensors suitable to monitor pH levels in highly complex 3D tissue models: (i) the biocompatibility of the overall system; (ii) the sensitivity in a wide-pH range of response along with 3D grafts; (iii) the non-invasiveness of the system [78].

Traditionally, on-line pH sensing for cell cultures has been carried out through different methods: electrochemical [79,80,81], ion-sensitive field effect transistors (ISFET) [82], light-addressable potentiometric (LAP) [69], or optical [83,84] detection methods.

#### 2.1.1. Electrochemical Biosensors

Electrochemical sensors are accurate and fast, but they require large sample volume and physical contact [85,86]. Moreover, conventional techniques (such as pH-meter probes or microelectrodes) provide only an average pH value, not taking into consideration the local discrepancies and thus the gradients which may be present in a 3D cell culture system. Novel devices such as microneedle sensors, able to pierce the sample without serious damages, have been developed, however they do not resolve the need for large volumes of culture medium, and are limited in design regarding shape, mechanical properties and sterility [87]. 

#### 2.1.2. ISFETs and LAPs 

On the contrary, ISFETs sensors can provide sensitive and repetitive measurements for small sample volumes of sample [88,89,90]; however, they still rely on physical contact. An ISFET device is normally composed of a metal-oxide-semiconductor field-effect transistor (MOSFET), where the metal gate electrode is replaced by a series combination of the reference electrode, electrolyte and chemically sensitive insulator, usually made in silicon. As a result, the culture medium is in direct contact with the insulator, which detects the ion concentration generating an interface potential corresponding to a current inside the semiconductor channel [91]. In this context, Lehmann et al. [92] demonstrated the use of ISFET-based sensors to explore the acidification degree in a 2D cell culture. 

Likewise, LAPs are semiconductor devices similar to ISFETs, widely employed in pH sensing for biological analysis [93,94]. The principle of operation of LAPs is based on a pH-sensitive electrolyte/insulator/semiconductor (EIS) structure. Usually, the insulating layer consists of silicon oxide and silicon nitride heterostructure, which separates the silicon substrate from the electrolyte. In this way, when hydrogen ions (H+) interact with the insulator, affecting the surface potential of the sensor, an activation of charge carriers by LED or laser is performed, causing a photocurrent, thus transducing the pH to an electrical quantity [95]. 

A recent work reported an efficient device to measure cancer cell metabolism in real-time. Authors developed a light-sddressable potentiometric sensor integrated with pH sensitive hydrogel nanofibers to monitor pH in breast cancer cell culture media in real-time, also in the presence of an anticancer drug [96]. Similarly, Yang et al. developed a fully integrated system, named Mirror-LAPS, which was able to easily construct 2D images and a real-time video to monitor the cellular metabolism and acidification of HK2 cells [97].

However, the use of LAP and ISFET sensors requires a physical contact, which currently limits their use in 3D cultures [64]. Moreover, most of the 3D scaffolds are composed of insulating materials, remarkably undermining their application in electrochemical detection [98].

Therefore, novel pH sensing approaches are required to adapt the biosensing techniques to hydrogel-based systems.

#### 2.1.3. Optical Biosensors

To date, several attempts have been directed towards the realization of optical pH sensors, largely due to their low costs, absence of immune and electrical interference, and non-invasive sensing also in a 3D environment [99]. Optical methods may be preferred over electrical counterparts since they do not require electrical connections and are less prone to electrochemical interferences induced by the biochemical species, allowing consistent and reliable surveys, also in 3D and dynamic environments [100]. 

Typically, the optical detection of pH is based on the measurement of the alterations in the optical property of a pH indicator [101].

In this respect, phenol red has been used as a stable biocompatible pH indicator, allowing the real-time inspection of acidification levels of the culture medium exploiting the different absorption characteristics of a culture medium enriched with phenol red, in accordance with its pH variations [86]. However, this technique detects the pH values in the surrounding culture medium and not within the 3D models.

The exploitation of fluorescence microscopy has provided additional techniques that can be used for probing the cell microenvironment pH in real-time with high spatial resolution.

Many fluorescent sensors have been recently developed to allow the analysis of pH alterations at a cell-size scale [102,103], as they represent a minimally invasive sensing technology, devoid of cell-damaging effects [104]. 

**Figure 1:**
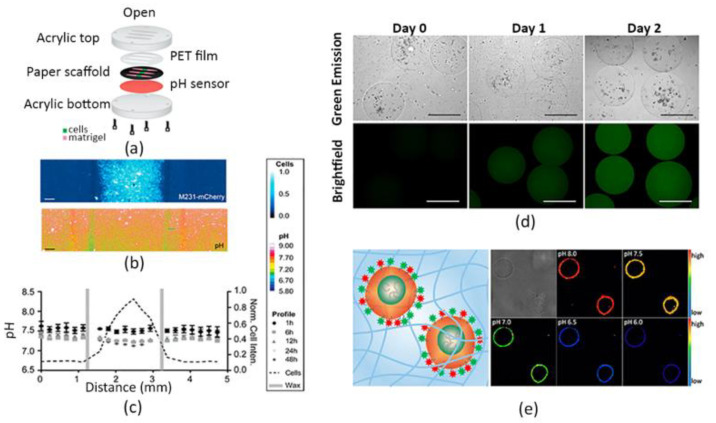
pH monitoring. (**a**) Representative illustration of the sensing culture platform containing the pH-sensing film assembled with a paper-based cell culture. (**b**) Fluorescence images of the engineered breast cancer cells cultured in delimited regions of the paper-based scaffold and corresponding heat maps showing the pH values spatial distribution. Scale bars are 250 μm. (**c**) Average pH profiles within the cell culture system over 48h, represented as mean and standard deviation of three different cell cultures. The dotted lines indicate the cells-seeded areas in the paper-based system. (**d**) Brightfield (top) and fluorescence images (bottom) of polyethylene glycol (PEG) microgels encapsulating HeLa cells and carbon dots pH nanoprobes over time; fluorescent signal intensity increasing over time indicate a decrease in pH level within the hydrogel cultures. Scale bar is 500 μm. (**e**) Representation of HeLa cells provided with surface-anchored lipid-DNA pH sensing probes embedded in a 3D collagen hydrogel (left); fluorescence signal emission in various pH extracellular levels. Signal intensifies with the increasing of pH values (right). (**a**–**c**) Adapted and reprinted with permission from [107]. Copyright (2018) American Chemical Society; (**d**) Adapted and reprinted with permission from [108]. Copyright (2017) American Chemical Society; (**e**) Reprinted with permission from [112]. Copyright (2014) American Chemical Society.

However, fluorescent molecules available for pH monitoring usually display intrinsic cytotoxicity and photobleaching effects. For example, fluorescein, which is widely used for detecting pH levels in the range of 6–7.2, can be easily photobleached [105,106]. To overcome this problem, Kenney et al. incorporated pH-sensitive fluorescein-based particles and pH-insensitive reference particles in a polyurethane thin film, thus creating a so-called “optode”. Subsequently, they placed these culture-compatible probes in a 3D tumor model containing MDA-MB-231 cells, as shown in Figure 1a, successfully measuring the spatio-temporal evolution of the extracellular pH gradients for 48 h in a range corresponding to that of normal and tumorigenic breast tissues (pH 6.5–7.5), as reported in Figure 1b,c. Importantly, the sensing platform revealed to be fast, not cytotoxic, reversible, as well as stable in a tumor-like structure, and evidenced only minimal photobleaching effects [107]. 

Other relevant results in monitoring pH values in 3D cell culture systems were achieved by Chandra et al., by encapsulating fluorescent particles within a 3D ECM-mimicking construct [108]. They reported the fabrication of 3D polyethylene glycol (PEG) microgels containing carbon dot-based pH nanoprobes as represented in Figure 1d, by employing a microfluidic assembly technique, to monitor time-dependent pH variations in the cellular microenvironment. Indeed, carbon-based fluorescent sensors are another class of fluorophores that have been reported by various groups for pH probing in cell cultures [109,110,111]. In the same paper, the authors preliminary demonstrated that the nanoprobes were biocompatible and that mammalian cells could be simply enclosed together with sensitive carbon dots in PEG gels. Further, they showed that microenvironment pH fluctuations in a physiologically-relevant range (5.8–7.7) can be revealed by using these 3D engineered hydrogels, suggesting that these systems are useful tools to analyze the cellular growth and disease progression [108]. 

Unfortunately, only few validated probes exist for the real-time detection of in situ pH within 3D ECM-mimicking models. 

Recently, in a paper published by Moldero et al., the basis for a new generation of smart pH sensing scaffolds has been established by realizing a cell-laden 3D scaffold integrated with capsules-based optical sensors aimed at investigating cellular microenvironment pH alterations during pathophysiological processes [87]. In particular, authors fabricated fluorescent micro-particle pH sensors based on the SNARF-1 fluorescent dye and entrapped them within a 3D printed scaffold, realized through the fused deposition modelling (FDM) technique. Human mesenchymal stromal cells were simultaneously seeded in the scaffold. In this way, they were able to detect time–spatial pH variations and gradient formation occurring in the in vivo-like microenvironment. Hence, they obtained a kind of pH map of the 3D culture system for seven days, differently from conventional methods that provide only bulk values without discriminating zonal variations [107]. Notably, the 3D cell-laden scaffold was also compared to the 2D respective system, revealing that pH changes were more evident in 3D conditions. Specifically, more acidic values were found within the scaffold with respect to the 2D culture, indicating that the presence of an ECM-like material influences the microenvironment surrounding the cells. 

In this context, Ke et al. developed a cell-surface-anchored fluorescent probe to measure the microenvironmental pH [112]. Specifically, the pH sensor consists of a lipid-DNA probe conjugated to the surface of cells, which were subsequently encapsulated in a type I collagen hydrogel. Authors reported that this system showed a sensitive and reversible response in the pH range of 6.0–8.0, as illustrated in Figure 1e, thus appearing useful for most pH extracellular analyses. Furthermore, confocal fluorescence imaging proved that entrapping these “opto-engineered” cells in the 3D matrix was an effective method to evaluate the acidification rate of the extracellular environment, opening new perspectives in the cellular metabolism investigation.

Up to date, only a few commercial optical pH sensors are available in the market [86]. However, they require physical contacts with the sample and suffer from dye leaching, thus reducing their use in complex 3D hydrogel-based cell culture. 

Therefore, many efforts are still needed to optimize existing pH sensors or design novel smart ones and to develop materials suitable for monitoring the pH fluctuations of the extracellular environment in 3D cell cultures. These devices should work in real-time for a long-term culture period, to generate spatial and temporal maps of the pH values throughout the 3D cell-seeded constructs in a highly reliable, repeatable, non-invasive and high throughput manner.

### 2.2. Glucose Biosensors

Glucose metabolism analysis is crucial for monitoring cellular status, as it represents the main energy source for cells activity.

Moreover, since glucose is a nutrient present in the culture medium and its concentration directly reflects the metabolic cellular state, it has always been considered as an indicative parameter of the metabolic activity of cultured cells [113,114]. 

A continuous monitoring of glucose in culture media has been conventionally performed to control and ensure the optimal environmental conditions in 2D cultures. To achieve this goal, different biosensing approaches have been developed. Interestingly, most of the devices currently employed in pre-clinical research derive from sensing tools used in the past years for self-measuring glucose levels in the blood by diabetic patients [115]. For instance, these include enzyme-based sensors that employ glucose-oxidase or glucose-dehydrogenase enzymes to evaluate glucose concentration in blood and interstitial fluids [116]. 

#### 2.2.1. Electrochemical Biosensors

Nowadays, electrochemically-coupled enzyme- and non-enzyme-based systems [117] have also gained popularity in cell cultures, because of their high selectivity and sensitivity, as well as low costs [118].

Among them, enzyme-based glucose biosensors have attracted more attention for the analysis of cell culture media. For example, a glucose-oxidase-based sensor exploits an oxidation reaction catalyzed by the glucose-oxidase. Notably, this enzyme is able to transform glucose in other metabolites with the production of hydrogen peroxide (H_2_O_2_), which, in turn, is amperometrically detectable through a working electrode. In this way, the chemically-induced current signal is directly correlated with the glucose analyte concentration [115]. Specifically, in 2D cultures, cells can adhere directly on the electrode surface for a continuous real-time detection. However, several researchers claimed that H_2_O_2_ accumulations damaged and caused adverse effects on cells [119]. To overcome this problem, sensors have been located far from cells, causing a detection only of the general bulk variations instead of precise local glucose concentration values [120,121]. 

In 3D models, the concentration fluctuations are smaller than in conventional cultures and are characterized by spatial gradients, making the detection of the analyte more difficult [64]. 

In this context, different research groups have successfully translated glucose biosensors, originally designed to work in 2D conditions, to 3D cell culture models by developing novel application-specific devices, to easily take advantage of the same glucose measurement principles exploited in 2D cell cultures in a more complex in vitro environment [122].

**Figure 2:**
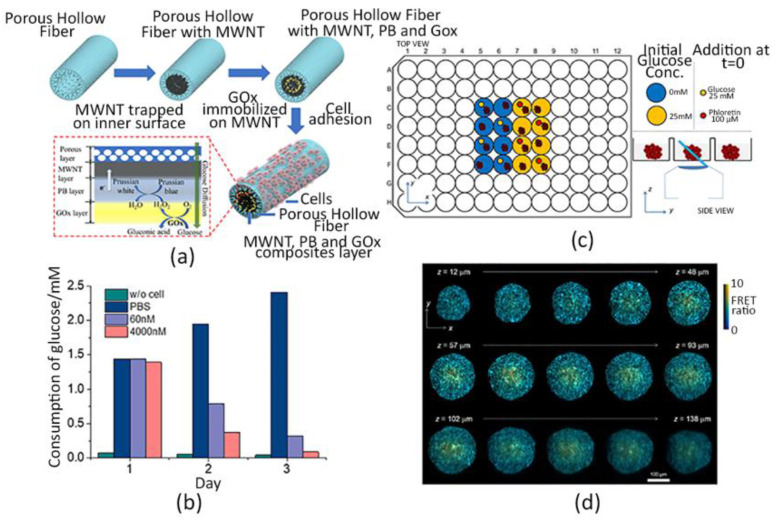
Glucose monitoring. (**a**) Schematic representation of the cellularized PHF scaffold integrated with the enzyme-based glucose sensor. Human lung cancer cells (PC9) adhere on the outer wall of the structure working as a permeable barrier for the glucose diffusion, whereas the enzyme is immobilized in the lumen. Here, electrochemical reactions occur through the sensing system composed of multi-walled carbon nanotubes (MWNT), glucose oxidases (Gox) and Prussian blue (PB). (**b**) Glucose consumption per day by PC9 cells cultured over the PHF upon different Osimertinib concentrations. (**c**) Scheme of the experimental set up: Matrigel-based HEK293T cells spheroids expressing the glucose FRET biosensor gene were seeded in a 96-well plate; different experimental conditions were tested and observed with the OPM technique, which is capable of orienting the light sheet towards the samples [130]. (**d**) Spatial glucose distribution within the Matrigel-coated spheroids at different depths (*z*-axis). Color scale and brightness determine the expressed FRET ratio and the emission intensity, respectively [130]. (**a**,**b**) Adapted and reprinted from [123], Copyright (2020), with permission from Elsevier.

For example, Ma et al. [123] realized a 3D hollow fiber structure provided with a glucose-oxidase sensing system. Specifically, they fabricated a polysulfone hollow fiber (PHF) composite, characterized by a peculiar gradient porous structure, where human lung cancer PC9 cells were grown onto the outer surface, whereas the enzyme was immobilized in the lumen, as shown in Figure 2a. In this way, the glucose sensor was located close to cells but not directly in contact, ensuring a continuous non-disruptive monitoring of the cell metabolism. In fact, experimental results showed higher sensitivity and stability of the sensor, also for H_2_O_2_ degradation, than conventional electrode structures. Their PHF system allowed to detect the glucose consumption vs. cell viability in response to 24-h exposure to two different concentrations of the anticancer drug Osimertinib, as shown in Figure 2b.

Another recent work about real-time monitoring of a metabolic function was published by Bavli et al. [124]. They fabricated a liver-on-chip device able to maintain HepG2/C3A cells embedded as spheroids in a collagen type I hydrogel for over a month under physiological shear stress and oxygen gradient conditions, closely resembling the liver-native scenario, to analyze the dynamics of mitochondrial dysfunctions. A commercial glucose-oxidase enzyme-based sensor was installed in a polymethyl methacrylate (PMMA) flow-chamber onto an off-chip switchboard controlled by a computer to provide automatic amperometric glucose measurements. Primarily, results revealed discrepancies in glucose concentration along the spheroid, caused by a different cellular consumption, thus mimicking the physiological glucose gradient experienced by cells in vivo. Further, together with O_2_ consumption, inn surveys performed by embedding O_2_ sensitive microbeads within the collagen hydrogel, authors showed that 62% of glucose was used in anaerobic glycolysis, showing the capability of this microdevice in monitoring metabolic changes in a clinically-relevant environment. 

However, even though enzyme-based sensors have already been commercialized, their employment has been restrained due to the chemical and thermal instability of the enzymes [118]. Hence, non-enzyme-based applications are being exploited to overcome these limitations. In addition, more versatile sensing approaches are required to work with 3D in vitro models. A considerable commitment to innovation is especially needed for hydrogel-based scaffolds, since they are made of low or non-conductive materials, thus hampering their adoption in electrochemical sensing. For example, one possible strategy may be to equip these insulated culture systems with conductive materials with electrocatalytic ability, as reported by Zhang et al. [98].

Another strategy was developed by Obregon et al. [118]; the authors took advantage of innovative nanomaterial-based films to fabricate a nanoporous gold (Au) biosensor to detect the glucose uptake of an in vitro model of skeletal muscle. Currently, the realization of a reliable pre-clinical model of this kind of tissue is of considerable significance, as it can be used both to evaluate drug efficacy against type 2 diabetes and to elucidate glucose uptake mechanisms during physical exercise, traditionally assessed through animal experimentation. For these reasons, authors cultured C2C12 myoblast cells on a micro-grooved gelatin methacrylate (GelMA) hydrogel, realized by utilizing a polydimethylsiloxane (PDMS) mold via the microcontact molding technique, thus creating an in vitro contractile muscle model. Next, in order to investigate the glucose consumption by the muscle, they synthetized a nanoporous Au (NPG) film, as described in their previous work [125], coupled with a three-electrode system to carry out electrochemical glucose measurements in the culture medium. The sensor showed a linear response in a wide concentration range [1–50 mM], thus demonstrating to be suitable for many glucose detections analyses. In addition, the measurements were performed in a rapid, real-time and non-invasive way, indicating that the nanomaterial-based approach is a relevant alternative to detect glucose uptake in engineered tissue-mimicking constructs, opening new scenarios for glucose monitoring in complex cell cultures.

Anyway, despite the aforementioned methods allowed to measure glucose uptake by adopting stable and non-invasive approaches in complex 3D cell culture architectures, they quantified the glucose concentration within the culture medium instead of detecting the 3D glucose spatial distribution within the ECM model. Moreover, culture medium components may influence electrochemical non-enzyme-based sensors, for example by occupying active sensing sites and consequently altering the measurement performances [118]. 

A more direct measurement of the glucose concentration in a 3D-cell encapsulation model was recently published [126]; in this work, glucose diffusion into a silated-hydroxypropylmethylcellulose (Si-HPMC) hydrogel containing human adipose-derived stem cells (hASCs) was investigated by the use of a micro-needle-based electrochemical glucose sensor. In particular, this sensor was implanted in the core of cylindrical-shaped cell-encapsulating hydrogels with different polymer concentrations and different numbers of seeded cells to examine the effects of these two parameters on the glucose diffusion in terms of cells viability. Glucose concentration analyses evidenced a reverse relationship between the number of viable cells and the polymer concentration, as well as cell density, indicating that the amount of available glucose within the gel strongly affects cell activity and viability. 

Nevertheless, in general, electrochemical sensors, such as the microneedle ones, have a limited lifetime and require a periodic recalibration [124]. Further, despite the work mentioned above, useful insights have been provided regarding the glucose transport within the hydrogel; the sensor employed was only able to provide a single-point glucose concentration measurement, in an invasive and potentially destructive manner for the polymer matrix. It was not possible to perform a spatio-temporal assay of glucose profile within the 3D model and to correlate it with the cellular spatial arrangement [126]. Moreover, although hydrogels are widely recognized as very promising materials for 3D culturing, little efforts have been made so far to map spatial and temporal gradients of glucose throughout 3D cell-seeded constructs, in spite of its relevance for cellular respiration and proliferation [126,127]. 

#### 2.2.2. Optical Biosensors

Only recently, a cutting-edge method has been developed by Maioli et al. which demonstrated that a time-lapse 3D imaging of multicellular spheroids (MCS), provided with a Forster resonance energy transfer (FRET) biosensor gene, may be a valuable approach to spatially monitor the glucose concentration within MCS. The genetically expressed FRET sensor is generally employed to measure distinct cellular metabolic-associated parameters, as its expression is directly related to the analyte concentration [128], whereas the use of 3D imaging techniques is used to show real-time cell activity, such as the light-sheet fluorescence microscopy (LSFM) [129]. Specifically, these authors applied the LSFM technique by adopting an oblique plane microscopy (OPM) to acquire time-lapse-3D images of the glucose FRET sensor within HEK293T cells spheroids embedded in Matrigel in 96-well plates, as shown in Figure 2c,d. Three experimental conditions with different glucose concentrations were tested. Interestingly, a spatio-temporal FRET ratio response map was generated for the three conditions, proving that an OPM-FRET coupling is a remarkable sensing system to record and display the 3D dynamics of cells activity in MCS over time [130].

However, this detection mechanism requires genetic modification which can impair gene expression. Thus, a possible strategy to overcome these limitations could be adapting detection technologies, currently used for measuring the culture medium glucose content, to the internal monitoring of the hydrogel. In particular, research should focus on sensing instruments capable of spatially mapping glucose concentration within cell-laden hydrogels to further elucidate the influence of nutrients distribution and exchange on cellular activity in vitro.

### 2.3. Oxygen Biosensors

One of the most important chemical cues to maintain cellular phenotype and function in vitro is surely the oxygen concentration, which is essential in the energy metabolism of the cells. O_2_ is a potent modulator of cell function and a transcriptional regulator for over 300 genes responsible for tissue homeostasis and maintenance [131,132]. Moreover, among the great variety of biochemical signals, O_2_ plays a pivotal role in modulating mammalian cell mechanisms both in healthy and diseased states [133]. Due to the physiological activities, O_2_ gradients naturally occur within biological tissues, affecting cell response and viability, as well as the preservation of tissue functions [132,134,135,136]. Furthermore, it is well-known that the correct O_2_ levels regulate different cellular mechanisms which are essential for cell differentiation [137,138,139,140].

Physiological O_2_ levels in the human body vary from ∼14% in lung alveoli down to ∼3% in muscle and skin [141]. Additionally, even lower values—around 0.5%—can be found in some tissues and are often correlated with a great variety of human pathologies, including cancer [142], tissues necrosis [143] and cardiovascular diseases [144].

However, an atmospheric O_2_ tension of 21% is generally present in cell culture models, affecting the reliability of the data obtained from in vitro culture systems and impairing their translation potential [145]. 

In this context, the proper implementation of systems capable of precisely reproducing and controlling physiological O_2_ concentrations in in vitro culture platforms is presently a critical challenge for organ modelling and tissue engineering.

As a consequence, different sensing strategies aimed at measuring and manipulating O_2_ profiles have been developed. 

The majority of sensors used to monitor O_2_ levels in 2D cell cultures are based on fluorescence quenching, which is an optical principle based on the evaluation of the fluorescence amplitude or the lifetime of a fluorescent dye. Generally, this is encapsulated in a sensor spot or spread in a polymeric membrane immobilized at the center of a well. Then, the fluorescence excitation and the luminescence emission readouts are performed non-invasively, by using LEDs or optical fibers [64]. Finally, the correlation between the sensor output and the O_2_ concentration can be found by considering the Stern–Volmer equation, which states that luminescence intensity is inversely proportional to O_2_ concentration [146,147,148]. 

#### 2.3.1. Electrochemical Biosensors

Electrochemical O_2_ sensors have been broadly employed to examine, for instance, the cellular respiration of adhering cells or to assess the pericellular O_2_ levels in the culture medium of 2D systems by using Clark-type or direct amperometric tools [149,150]. In both cases, the working principle is based on the reduction of O_2_ at the level of a noble metal electrode. Nevertheless, while the cell culture medium is directly in contact with the electrode in amperometric sensors, a gas-permeable membrane separating an independent sensor electrolyte from the medium solution is present in Clark-type tools. This specific configuration was implemented, since contaminations of the electrode surface by cell adhesion or protein adsorption can occur in non-Clark-type designs, resulting in a lower specificity and stability of the sensor [151,152].

However, it is necessary to adapt the previously mentioned sensing approaches to monitor O_2_ levels also in 3D biomaterial-based models to establish optimal environmental conditions necessary to ensure the correct cell functions [153,154]. 

One of the first and traditional methods for O_2_ probing in 3D culture platforms was proposed by Weltin et al., who positioned an electrochemical two electrodes-based microsensor close to a spheroid composed of hepatocytes exposed to an anticancer drug within a 96-well plate. In this way, authors combined information related to the real-time O_2_ consumption of the cells with toxicological data, indicating how metabolic alterations are correlated with increasing levels of drug exposure. However, only a spheroid global quantification of the O_2_ present in the medium was performed by using this methodology, without providing information about the oxygen concentration throughout the 3D spheroid [155]. 

#### 2.3.2. Optical Biosensors

**Figure 3:**
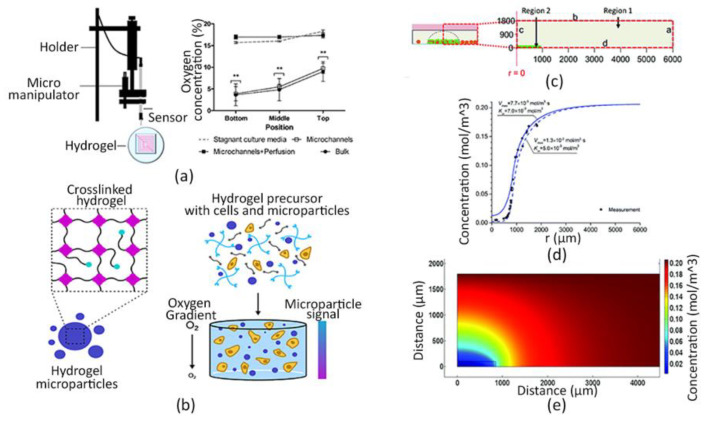
O_2_ detection. (**a**) The optical fiber-based sensor set up composed of a micromanipulator and an optical needle microsensor to investigate O_2_ concentration within 3D cell-laden hydrogel (left); O_2_ levels measured at different depths (bottom, middle position, top) after 24h of bioprinting in the following tested conditions: “Microchannels”, indicating the presence of microchannels within the hydrogel without perfusion; “Microchannels + Perfusion”, indicating the presence of microchannels within the hydrogel with perfusion; “Bulk”, indicating the absence of microchannels within the hydrogel. Stagnant culture media were used as control (right) ** *p* < 0.01 when compared to culture media [156]. (**b**) Schematic illustration of the 3D millimeter-scale cellularized PEG hydrogel: hydrogel-based microparticles biosensor incorporating O_2_-sensitive fluorescent dyes (left) were encapsulated within cell-seeded PEG hydrogel (right). (**c**) Side-view representation of the cell-free (Region 1) and the cell-laden (Region 2) collagen hydrogel. Polydimethylsiloxane PDMS O_2_ sensing microbeads were distributed in the entire polymeric matrix. Dotted lines delimit the computational domain. Boundaries “a” and “d” represent PDMS– and polystyrene–hydrogel interfaces, respectively. Boundary “b” represents cell culture media–air interface. Boundary “c” represents the z-axis at a radial position of 0, in the disk-shaped hydrogel. Regions 1 and 2 represent the cell-free hydrogel plus cell culture media, and cell-laden hydrogel regions, respectively. (**d**) Experimental data of O_2_ concentrations and their best fitting within the 3D hydrogel versus the distance from the center of the hydrogel. (**e**) Spatial map of the simulated O_2_ concentration within the selected computational domain. (**b**) Adapted and reprinted with permission from [153]. Copyright (2019) American Chemical Society; (**c**–**e**) Reproduced from Ref. [169] with permission from the Royal Society of Chemistry.

Recently, bioengineering research has progressed to tackle the integration of optical sensors, able to spatially monitor O_2_ concentration, with 3D hydrogel-based systems. Figueiredo et al. evaluated the O_2_ concentration within a cell-laden hydrogel provided with a perfusable microchannel network by using a needle-type optical fiber microsensor (PreSens, Regensburg, Germany) and a micromanipulator (Eppendorf TransferMan NK2, Hamburg, Germany) (Figure 3a). O_2_ levels were detected for 24 h at three different depths within the hydrogel to investigate whether the presence of a perfusable network, mimicking tissue vascularization, could affect cell viability [156].

Although this sensing method is simple, effective and gives preliminary insights about the O_2_ concentration profiles in 3D cell-seeded constructs, its data output is too limited to map the 3D spatial distribution of the O_2_ throughout the model. Moreover, it is an invasive method, being the needle diameter in the range of 200–250 μm, which can lead to irreparable damages of the samples [157]. 

For these reasons, it is necessary to design other types of optical O_2_ sensors capable of mapping spatial–temporal variations and gradients in 3D cell-laden scaffolds without affecting the whole system, and possibly providing real-time quantitative monitoring of O_2_ concentration with a sub-cellular spatial resolution. Hence, further optical technologies have recently gained great attention, since they may offer a wide spectrum of versatile bioengineering tools suitable for 3D in vitro models [136,158,159]. 

In this scenario, Rivera et al. integrated a photonic O_2_ biosensor into a 3D tissue scaffold. The biosensor is a phosphorescence-based O_2_ sensor that employs the quenching of palladium-benzoporphyrin to transduce the local O_2_ content, and it was validated by using both healthy and tumorigenic breast epithelial cells, MCF-10A cells and BT474 cells, respectively, cultured under normoxic and hypoxic culture conditions [160].

Similarly, Boyce and colleagues prepared a polystyrene thin film containing palladium tetrakis(pentafluorophenyl)porphyrin (PdTFPP) molecules, whose luminescence is quenched by O_2_, and placed it in contact with a cellulose scaffold containing a Matrigel hydrogel encapsulating MDA-MB-231 cells to measure spatial and temporal O_2_ gradients within the culture. Oxygen diffuses into the polystyrene thin films, resulting in variations in luminescence intensity. The sensor is compatible with optical- and fluorescence microscopies, sensitive to small changes in oxygen tension. Authors observed oxygen gradients formed in paper-based scaffolds containing fluorescent breast cancer cells. Consequently, the O_2_ consumption rate of cells was analyzed by simultaneously visualizing the luminesce intensity of the O_2_-sensing film and the fluorescently labelled cells [161].

In another work, the cells-embedding hydrogel and the same sensing film were incorporated by Boyce and colleagues in a Block-Layered O_2_-Controlled Chip (BLOCC), which is a modular multi-layer device realized by assembling multiple alternate acrylic and silicone layers. In particular, this chip was designed to have cell-containing chambers in the center between two lateral parallel channels, where oxygenated and deoxygenated gas mixtures were flowed to impose physio-pathologically-relevant O_2_ gradients in the cell-seeded regions. O_2_ gradients and resulting cellular responses were simultaneously mapped in real-time to examine whether these cells modify their activity proportionally to O_2_ tensions [162]. 

Nevertheless, the three-dimensionality of the matrix was not entirely mapped by the film, therefore limiting the O_2_ investigation to a single plane. 

To overcome this issue, a possible alternative was developed by Wolff et al., by integrating an O_2_ probing foil with an additional optical fiber-based sensor set up, to display the oxygenation level within the construct along the *z*-axis of the scaffold. In this way, the identification, localization, and temporal observation of the O_2_ dissolved within the 3D cell-loaded hydrogels was performed [163].

However, this micro sensor is invasive, impairing the integrity of the hydrogel. Moreover, this approach requires the use of two different O_2_ sensors and thus the integration of two different data sets, resulting in a very laborious and expensive procedure. 

For these reasons, more recently, several research groups have proposed the combination of O_2_-sensitive fluorophores with micro- or nano-particles as a potential alternative to O_2_ sensing films for monitoring O_2_ concentration in 3D hydrogels [164]. In particular, carboxylic acid-modified polystyrene nanoparticles (NPs; 510 nm in diameter) were functionalized with a commercially available O_2_-sensitive fluorophore for measuring the O_2_ gradients in different cellularized hydrogel-based environments. These custom-made fluorescent nano-O_2_ particles (FNOPs) were incorporated inside electro-sprayed calcium (CaCl_2_)- and strontium (SrCl_2_)-gelated alginate beads (700–1000 µm in diameter) containing HeLa or RIN-m5F cells to fabricate a model of the pancreas in vitro. This sensing culture system showed a good dynamic range and resolution as well as the capability to show the 3D distribution of the analyte within the 3D hydrogel-based tissue by using fluorescence microscopy. Higher cell viability in the external areas of the hydrogel compared to the inner region was found, due to the establishment of O_2_ gradients through the hydrogel, attributable to the well-known gas limitations in diffusion. Moreover, results reported first a decrease in O_2_ concentration within the hydrogel due to the cells consumption, and then a reverse increased analyte tension, probably due to the exponential decay of the cellular density within the gel over time [165].

Likewise, Wilson and colleagues developed fluorescent hydrogel microparticles to monitor O_2_ levels in 3D artificial tissues, up to 2 mm in thickness, which did not cause any cytotoxic effect and displayed an excellent photostability. In particular, these microparticles contained an O_2_-sensitive porphyrin dye and an O_2_-insensitive reference dye, to avoid photobleaching. They were synthetized from an organic-in-oil suspension, and then embedded in mm-scale cellularized PEG hydrogels, as shown in Figure 3b. O_2_ gradients in physiological ranges were accurately detected across the entire polymeric matrix and, notably, the O_2_ consumption of both primary and transformed cells within the 3D in vitro model was measured by combining the experimental methodology with a computational one [153]. In fact, in silico models can help researchers in the fabrication of clinically-relevant in vitro models by predicting the nutrients and O_2_ delivery and waste products removal kinetics within size-relevant tissue-engineered scaffolds [154,166,167,168].

Pérez et al. combined dispersible O_2_-sensing PDMS microbeads and computational fluid-dynamic mass transport simulations to estimate the O_2_ uptake rate of breast cancer cells encapsulated within a collagen hydrogel. Specifically, MDA-MB-231 cells were dispersed only within a specific region of a 3D collagen hydrogel, whereas the microsensors were entirely distributed within the hydrogel to consider two different areas: a cell-free region surrounding a cell-laden one (Figure 3c). O_2_ concentration was assessed in both areas by using phase fluorimetry, which is a method exploiting the luminescence lifetime of an indicator. Then, O_2_ values were analyzed producing spatial graphs of the analyte distribution, which highlighted significant discrepancies in the O_2_ microenvironment between the two regions, as illustrated in Figure 3d. Lastly, a computational model of O_2_ supply, diffusion and consumption was implemented to calculate the O_2_ uptake rate and the half-saturation constant of MDA-MB-231 cells by performing the best fitting of the O_2_ profiles experimentally observed (Figure 3e) [169]. 

The advances here reported have highlighted the importance of integrating non-invasive, easy-to-use and biomaterial-compatible O_2_ sensors. In particular, important information regarding the crucial role of O_2_ for cell activity and viability were achieved by using fluorescent-based technologies [170,171,172].

Among the different typologies shown, the micro/nano-particles based-ones seem to better fulfill the desired requirements, having the capability to perform real-time measurements of the O_2_ profiles throughout the entire three-dimensionality of the culture model. The employment of such O_2_ detecting systems can represent a significant advancement towards the development and translation of viable and controllable organ-scale constructs.

## 3. Biosensors to Monitor the Cellular Behavior: Impedance Biosensors

### 3.1. Working Principle

Impedance biosensors are some of the most important cell-based biosensors currently available since represent an automation-compatible label-free technology which enables to obtain data in real-time on the cellular conditions. In particular, cellular impedance biosensors are capable of monitoring cellular viability, adhesion and spreading, for any adherent cell type, by monitoring electric variations at the contact surface between the cell and an electrode [173].

They are typically developed by immobilizing a group of cells on an array of electrodes onto an insulated substrate for real-time data acquisition, analysis, and display. 

In the past years, cellular-based impedance sensing has been adopted for real-time, non-invasive, and non-disruptive monitoring of cell viability [174,175,176,177,178,179].

Programmed cell death, or apoptosis, which is the desired therapeutic response of a cancer cell to effective chemotherapies or radiation treatments, is characterized by dramatic changes in cell morphology, ionic channel conductance, and extracellular membrane integrity, as well as altered intracellular structure [180,181,182]. These electrical property variations can be diagnosed by cell impedance biosensors at significantly reduced costs and through expedited assessment procedures [173].

In particular, to measure the electrical variations of a cell culture, a frequency-dependent small sinusoidal voltage (V(ω)) is often applied through the cells (or tissue) and the variations in the resulting sinusoidal current (I(ω)) between the electrodes is recorded [183,184,185]. Hence, the electrical impedance (Z(ω)) of cell culture is derived using Ohm’s law. More specifically, when a sinusoidal voltage (typically at 10 kHz) is applied on interdigitated electrodes, an ion current is formed between the electrodes. As soon as cells grow and attach to the conductors, the impedance will increase since cells behave as non-conductors at low frequencies. Conversely, when cells die, they detach from the electrodes and the impedance decreases [186].

So far, several circuits have been designed to monitor the electrical properties of cells grown in 2D systems, due the aforementioned correlation between the electrical signal outputs and changes in cell activity [183,184,185,187]. 

However, the use of impedance biosensors in 2D cultures relies on cells attaching and spreading directly over the electrodes. This does not occur when cells are embedded within 3D hydrogels, therefore there is an urgent need to adapt these biosensing tools to a 3D context.

In general, it is mandatory to ensure a continuity of the electric field through the whole 3D system and to maintain unaltered mechanical properties of the hydrogel in spite of the presence of the electrodes in the culture systems.

### 3.2. Impedance Biosensors

**Figure 4:**
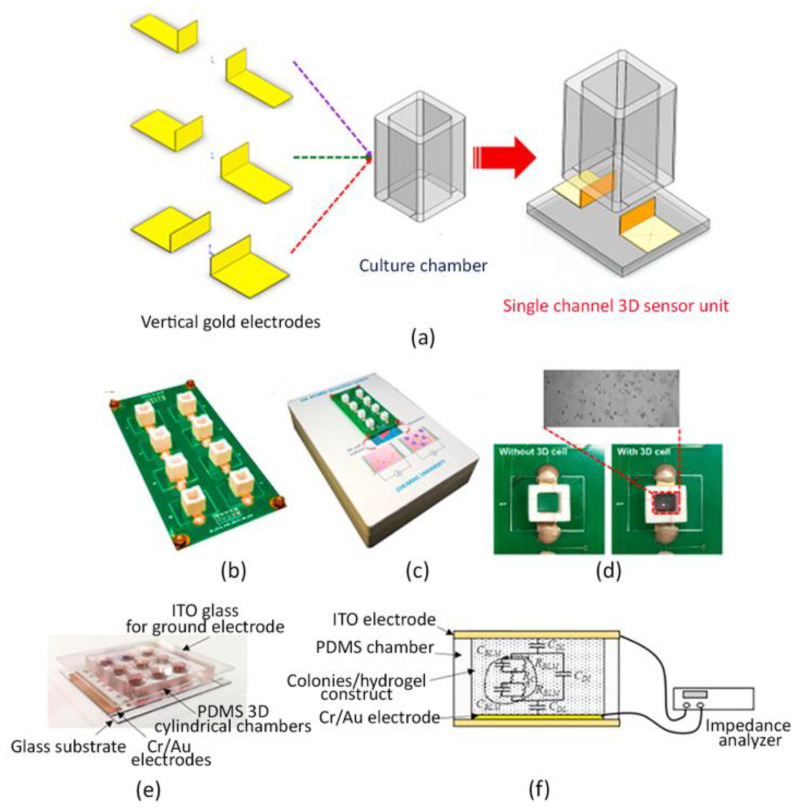
Impedance sensors. (**a**) Scheme of the 3D impedance biosensor single-unit composed of two vertical gold electrodes assembled with the PET culture chamber and the glass substrate. (**b**) Eight-channel 3D impedance biosensor. (**c**) 3D electric cell/matrigel-substrate detection platform containing the 3D impedance biosensor, a signal-conditioning module and a computer-controlled data acquisition card. (**d**) Illustration of the 3D single impedance biosensor before (left) and after (right) culturing HepG2 embedded in Matrigel hydrogel. (**e**) Image of the multi-layer impedance sensor consisting of an indium tin oxide (ITO) glass slide for the ground electrode, a PDMS layer composed of nine independent 3D cylindrical chambers able to host 3D cell-laden hydrogels, and a glass substrate provided with nine Cr/Au electrodes. (**f**) Illustrative design of the experimental set-up and the equivalent circuit to monitor impedance in cancer colonies within 3D hydrogels between the parallel plate electrodes. (**a**–**d**) Reprinted from [188], Copyright (2019), with permission from Elsevier; (**e**,**f**) Reprinted from [190], Copyright (2015), with permission from Elsevier.

Recently, an electric cell/matrigel-substrate impedance sensing system (3D ECMIS) was developed. Specifically, the 3D ECMIS consisted of eight individual sensor units, each of which composed by a pair of vertical electrodes, a culture chamber and a glass substrate, as represented in Figure 4a. The Au electrodes were laser-cut and subsequently attached to the inner surface of a polyethylene terephthalate (PET) culture chamber. Moreover, an eight-channel 3D ECMIS detection system was incorporated to record the impedance output signal (Figure 4b,c). As shown in Figure 4d, a model of liver cancer was realized by embedding human hepatoma cells (HepG2) within a 3D matrigel matrix, and the efficacy of three different anti-cancer drugs was tested by using the impedance sensing platform previously assembled. Finally, results were compared with traditional fluorescent staining. Authors reported increased impedance values with time in respect to the cell-free Matrigel control, meaning that the cellular growth within the hydrogel-based system conditioned the impedance of the system. Moreover, the use of anti-cancer drugs led to a major impedance decay. Interestingly, the impedance biosensing system exhibited a high consistency with the conventional imaging method for monitoring 3D cell viability, showing that this approach can represent a promising platform for 3D-culture cell-based drug screening [188].

In another recent work, a methodology for the high throughput and quantitative drug screening of cells cultured in the 3D environment was proposed by combining a paper-based cell culture model with impedance sensors. A paper substrate is a reticulated structure obtained by patterning a paper substrate (i.e., a filter paper) to obtain an array of culture wells. More specifically, cells embedded in a 3D agarose hydrogel were cultured over an array of circular culture microwells printed on a paper substrate to realize multiple hydrogels in a highly reproducible manner. Cell-containing agarose was gelled directly into the microwells of the paper substrate, which was subsequently submerged in the culture medium, containing different substances under testing. Measurement of impedance values was based on a setup of a three-electrode system, fabricated over a glass substrate by standard micro-fabrication processes, including chrome (Cr)/Au deposition, photolithography, and metal etching, assembled. The paper substrate allowed the easy transfer of the 3D hydrogel-containing cells from the culture platform to the detection one. In fact, during measurements, it was placed over the glass substrate and the microwells were precisely positioned onto the electrodes. In order to assess the impedance of the culture system, an electric potential of 0.1 Vrms was applied across the electrodes and an impedance analyzer recorded the measurements. Such values were reported to be harmless for the cells suspended in the hydrogel. Subsequently, the impedance magnitudes measured from 100 Hz to 100 kHz were collected and correlated to cell viability; the latter was calculated as the percentage of live cells between the control impedance magnitude (100% live cells in the construct) and the background one (hydrogel only). In addition, to demonstrate the feasibility of drug screening tests by using this sensing system, cell viability of two human hepatoma cell lines (Huh7 and Hep-G2) treated with two drugs (doxorubicin and etoposide) was evaluated. The results showed that Huh7 cells had a higher drug resistance than Hep-G2 cells and doxorubicin had higher efficacy than etoposide for treating hepatocellular carcinoma, as confirmed by simultaneous fluorescence image analysis of cell viability [189]. 

Impedance sensors have been also successfully adopted to monitor the formation of colonies of cancer cells. In particular, the colony formation assay is considered the gold standard to assess the development of early tumors in vitro and, eventually, to evaluate the effects of cytotoxic agents on their growth.

However, conventional colony formation assays are based on manual counting of the generated colonies under a microscope, which is laborious and not accurate. 

Interestingly, in a recent work, an impedance-based biosensor was developed to quantify the cancer cell colonies suspended in a 3D hydrogel. A human hepatoma cell line, Huh-7, established from a hepatocellular carcinoma, was used and the chemosensitivity of cancer cell colonies under different concentrations of an anti-cancer drug—i.e., doxorubicin—was quantitatively investigated. A biosensor embedded with a pair of parallel plate electrodes was developed for the impedimetric quantification of the cancer cell colonies. In particular, the biosensor consisted of three layers, including a glass layer for common ground electrode, a PDMS layer for the independent cylindrical chambers, and a glass substrate with 9 Cr/Au measurement electrodes, as represented in Figure 4e. In such a configuration, the colony formation process was quantitatively assessed by deriving a colony index from the measured impedance magnitude (Figure 4f). 

In addition, the colony size was also monitored by analyzing the phase angle of impedance. Notably, as demonstrated by the authors, the knowledge of both parameters provided quantitative and objective information to describe the formation and size of cancer cell colonies suspended in a 3D system, allowing an efficient, relatively faster and simple method for the quantification of cancer cell growth [190]. 

In summary, impedance-based biosensors are label-free and non-invasive tools to monitor the status of cells cultured in 3D hydrogel-based environments. However, in 3D cultures, cellular processes are more complex than in 2D culture conditions, thus impedance variations may be affected by different factors such as hydrogel matrix degradation and cell migration. Therefore, the integration of these categories of biosensors with other label-free and real-time monitoring systems is required to allow a full multiparametric approach to monitor the cell activity in 3D environments.

## 4. Biosensors to Detect Secreted Molecules

Cellular behavior is closely related to a wide number of cells-secreted molecules, which regulate cell proliferation, migration, cell–cell signaling and cell–ECM interactions. It is important to detect the activities of functional molecules released from cells, under different culture conditions to investigate fundamental aspects of cell biology and to establish innovative therapies aimed at targeting biological pathways in pre-clinical studies [191]. 

Indeed, a significant variation in molecular biomarkers levels may be indicative of critical changes in natural tissues physiology, indicating, for example, the origin of a tumor [192]. Moreover, different types of secreted proteins have been described as pivotal modulators of several cellular mechanisms, such as differentiation or communication [193,194,195].

For example, cytokines and chemokines have always been of great interest among researchers, since these small proteins are especially involved in the onset and regulation of immune responses. In fact, they affect every step of the immunomodulation process, such as the reproduction, recruitment and efficacy of immune cells during inflammation and attacks of pathogens [196,197,198].

To date, the most common techniques employed to examine cytokines profiles rely on flow cytometry, enzyme-linked immunosorbent assay (ELISA) and enzyme-linked immunosorbent spot (ELISpot) assays [199,200,201,202,203].

Despite this, their application for the detection of target biomolecules in in vitro models is flawed. In particular, flow cytometry is usually employed to evaluate the percentage of cells within a sample producing a specific marker, but it cannot estimate the number of molecules secreted by cells in the extracellular environment [204]. On the other hand, ELISA and ELISpot are well-established tools to investigate cell-secreted proteins via antibody (Abs)-antigen binding. However, they are prefabricated commercial systems which allow the detection of a single or multiple kind of molecules, but real-time information about secreting pathways cannot be simultaneously provided, since these assays can be performed only at single time points [205].

Conventionally, other protein detection systems are based on surface plasmon resonance (SPR) [206], mass spectrometry (MS) [207] and surface-enhanced Raman spectroscopy (SERS) [208]. Nevertheless, they are very expensive, labor intensive and time-consuming [205,209,210].

For these reasons, there is an increasing interest in developing new detection tools capable to provide more detailed information about the kinetics of cell-released molecules, with more rapid and gentle protocols for cell culture manipulation, also reducing working volumes and costs [211].

### 4.1. Antibody-Based Biosensors

Microfabrication techniques have been largely employed to realize monitoring platforms characterized by well-organized patterned surfaces of self-assembled monolayers, which allow the firm anchoring of Abs able to capture specific ligands of interest [212,213,214].

In particular, gold (Au), silicon, glass or PEG-coated glass slides have been functionalized with Abs spot microarrays for cells attachment or specific molecules binding sites. Traditionally, the most common methods of protein patterning include photolithography and soft lithography technologies. Briefly, they consist of transferring a geometric pattern to a light-sensitive substrate by using a photomask and ultraviolet irradiation, or an inked elastomeric stamp placed in contact with the surface, respectively. Furthermore, conventionally, PDMS or PEG microwells are frequently fabricated to confine cells in multiple small areas, as close as possible to the sensing domain, in order to improve their sensitivity and selectivity [215,216,217,218,219].

Hence, these novel devices allow simultaneous exploitation of the high specificity of Abs-based assays, such as ELISA and ELISpot, to significantly reduce the amounts of reagents and costs, as well as to get multiple real-time measurements, thus remarkably improving the accuracy of the acquired information [191,211,220].

Adapting these current sensors to the emerging 3D biomaterial-based in vitro models is necessary to precisely monitor small messenger molecules secretion in highly realistic in vitro environments [100,221].

With this purpose, Berthuy et al. fabricated a biosensing system composed of an Au-slide provided with specific Abs spots for the real-time identification of prostate-specific antigen (PSA), involved in the phases of prostate cancer, and β_2_-microglobulin (β_2_M) released by a human prostate carcinoma cell line (LNCaP) embedded in 3D alginate hydrogels under dihydrotestosterone (DHT) stimulation. More specifically, alginate beads were realized over the Au substrate placed within a culture chamber, so that the cells could grow in a 3D environment directly in contact with the sensing domains. Subsequently, a SPR prism was integrated below the chamber and the assembled system was inserted into a SPR imaging device. This tool assessed the PSA and β_2_M levels by measuring the refractive index variations of a polarized light exciting the Au surface, since these changes can be correlated with the molecules adsorption or desorption from the sensing regions. In this way, authors showed the different release kinetics of these two proteins after only 20 min, differently from the traditional ELISA assay, which cannot effectively detect these molecules within 24 h [222].

Among other sensing principles currently employed, electrochemical-based approaches offer remarkable stability, sensitivity and biocompatibility, as well as the capability to perform long-time analyses [223,224]. Furthermore, electrochemical sensors are particularly suitable to be incorporated within microfluidic devices, due to their simple fabrication and miniaturization [224]. Notably, they can be efficiently functionalized with immobilized Abs on their surfaces to exploit specific binding for targeted biomarkers [221,224].

For instance, Shin Su Ryon et al. have recently provided Au electrodes with an Abs binding self-assembled monolayer to realize a microfluidic electrochemical biosensor. This system was connected to a micro-bioreactor hosting GelMA hydrogel embedding human primary hepatocytes to evaluate the acetaminophen effects on liver cells, by monitoring albumin and glutathione-S-transferase-alpha (GTS-α) production, as they represent crucial indicators of hepato-toxicity. Multiple online measurements were carried out and revealed that results were comparable with those collected with conventional ELISA assays, proving the ability of the platform to precisely detect cells-secreted molecules in a 3D dynamic environment [204]. Subsequently, the same electrochemical technology was also successfully adopted in a dual organ-on-chip platform, where GelMa-based organoids mimicking the liver and heart were cultured to simultaneously assess drug induced hepatotoxicity and cardiotoxicity by monitoring albumin, GST-α and creatine kinase-MB soluble biomarkers, respectively [225]. Similarly, they utilized the same approach in a further implementation of a multi-organ system that also included a lung organ model [226]. Interestingly, another group has recently developed a bioprinted muscle model that was stimulated both electrically and biologically. The sensing system, composed of Abs-functionalized screen-printed gold electrodes, was fluidically connected to the organ compartment for the detection of IL-6 and TNF-α upon tissue stimulations [227].

Although Abs immobilization on the sensor surface is typically used to detect cell- secreted markers within microfluidic devices [228,229,230,231], one of the main disadvantages of this approach is the fast saturation of the sensing systems, which limits their application for continuous long-time analyses [232].

To overcome this issue, reconfigurable devices or an additional washing system can be implemented and integrated, but these ameliorations still remain challenging and labor-intensive [233,234]. Alternatively, microbeads can be coated with Abs and directly inserted and removed within microfluidic channels, as suitable substitutes to static biosensors. In fact, when particle surface saturation occurs, the sensing system can be rapidly regenerated by merely injecting a new set of beads within the device without the need for any additional steps of washing [204,235,236].

For example, Riahi Reza et al. combined Abs-coated magnetic microbeads with an electrochemical sensing microfluidic chip to investigate the effect of different acetaminophen concentrations onto albumin and transferrin release by human hepatocytes. Specifically, liver spheroids embedded in 3D GelMa hydrogels were placed within a bioreactor fluidically connected to the chip. In particular, the microdevice was designed with two chambers provided with a magnet for microbeads immobilization during the antigen detection, and an electrochemical microelectrode for signal production. Moreover, a computer-controlled microvalve system was implemented within the chip to allow automatic loading and replacement of the magnetic microbeads after each measurement, as well as transferring of sample solutions from the liver bioreactor to the sensing platform. The process could be efficiently repeated by releasing the magnetic field and activating specific valves, flushing out microbeads and samples volumes into separate reservoirs, and immediately starting a new cycle of measurements. Indeed, this platform allowed long term assays to be carried out, precisely verifying the changes of the hepatic cell-secreted biomarker levels upon drug treatments. Results turned out to be comparable with those obtained with standard ELISA control assays, thus demonstrating the accuracy of the sensor performance [232]. 

**Figure 5:**
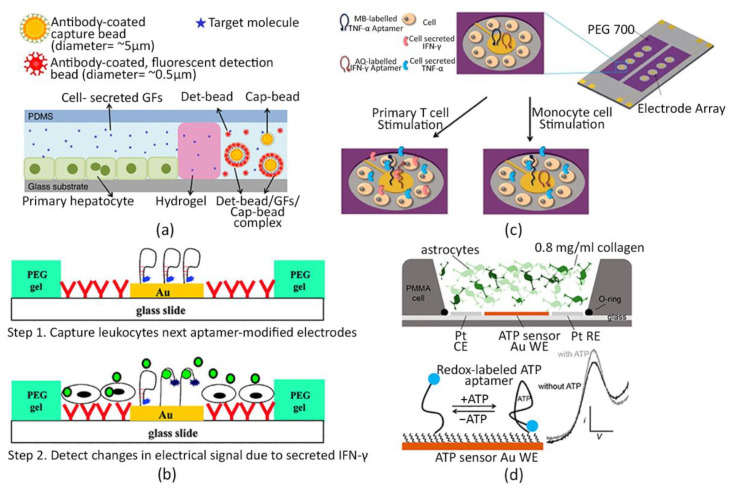
Cell-secreted molecules detection. (**a**) Illustration of the microfluidic chip composed of two chambers for human primary hepatocytes cultivation and cell-produced growth factors (GFs) quantification, respectively. The chambers are separated by a 3D PEG hydrogel barrier, which allows cell-released molecules to diffuse towards the sensing region, where polystyrene Ab-modified microbeads are injected to monitor local GFs concentrations [237]. (**b**) Scheme of the aptasensor design. Aptamer-modified Au electrodes are fabricated on glass slides provided with T-cell-specific Abs. PEG hydrogels surrounding the electrodes define cell attachment sites in the proximity of the sensing domains (top); T cells are captured on Ab-functionalized glass regions next to the aptasensors which detect leukocytes-released IFNγ (bottom). (**c**) Arrays of Au microelectrodes modified with a mixture of aptamers for IFNγ or TNFα binding labelled with thraquinone (AQ) and methylene blue (MB), respectively. T-cells or monocytes are bound next to aptasensors, changing their conformation after cytokines binding. (**d**) Cross-sectional image of the sensor/3D cell culture set-up. A commercial PMMA holder assembled on a glass surface contains a three-electrodes system directly interfaced with a 3D collagen hydrogel for astrocytes cultivation and stimulation inducing ATP production (top); Aptamers immobilized on the sensing electrode surface switch their structure upon ATP binding, resulting in a quantitative electrical signal change (bottom). (**b**) Adapted and reprinted with permission from [258]. Copyright (2011) American Chemical Society; (**c**) Adapted and reprinted from [260], Copyright (2015), with permission from Elsevier; (**d**) Reprinted with permission from [272]. Copyright (2019) American Chemical Society.

Similarly, Son Kyung Jin and colleagues realized polystyrene Abs-modified fluorescent microbeads and infused them into a microfluidic chip to monitor hepatocyte growth factor (HGF) and transforming growth factor (TGF)-β1 secretion. Specifically, primary hepatocytes were grown within a first chamber as a monolayer adjacent to a 3D PEG hydrogel structure, mimicking the native ECM barrier, through which released HGF and TGF-β1 could spontaneously diffuse towards a separate chamber; here, fluorescently-labelled microbeads functionalized with anti-HGF and anti-TGF-β1 were injected (Figure 5a) and the quantitative measurements of each cytokine concentration were performed. Consequently, their chip design allowed to detect the local concentrations and secretion rate of HGF and TGF-β1 without disturbing cell activity for seven days [237]. 

### 4.2. Aptamer-Based Biosensors

Valid alternatives to Abs-based biosensors in cells culture application are based on the use of aptamers, which consist of DNA or RNA strands [238,239,240,241,242,243] that have already been adopted as sensing elements to effectively detect a large variety of molecular biomarkers [244,245,246,247,248,249,250].

Indeed, aptamers offer a better thermal and chemical stability, as well as a more stable sensibility over environmental perturbations, if compared to Abs-based sensors [251]. Moreover, they are characterized by a simpler molecular structure than Abs which can be easily modified with functional groups [252,253] and designed into beacons, which directly emit optical or electrical signaling once the targeted analyte is bound, without the need for further labeling or washing steps [247,249,254,255,256,257]. 

In this context, Liu et al. largely employed aptamer-based microfluidic immunosensor for monitoring local interferon gamma (IFN-γ) release from primary human leukocytes. Authors designed an Au microelectrode array assembled on a glass surface; the microelectrodes were functionalized with aptamers labelled with a redox probe. Cell attachments sites were located immediately next to each electrode, as represented in Figure 5b. Then, these micropatterned slides were integrated within a PDMS microfluidic device where blood samples were infused to capture CD4+ T cells and evaluate IFN-γ production. Since IFN-γ is a fundamental inflammatory cytokine correlating with T cells immunological response to several diseases, such as human immunodeficiency virus or tuberculosis, a further development of these biosensors could provide robust support to the immunological research and diagnostics [258]. 

Subsequently, the same authors improved this device to simultaneously assess the presence of IFN-γ and tumor necrosis factor (TNF)α secreted by both T cells and monocytes, by adopting two different novel configurations: (i) a single electrode functionalized with a specific aptamer for IFN-γ or TNFα [259]; and (ii) a single electrode provided with both specific aptamers for IFN-γ and TNFα (Figure 5c) [260]. 

Moreover, in a further work, authors modified the microfluidic chip design with a reconfigurable one to study the paracrine cross-talk via TNFα signaling between two groups of cells. To this aim, the microfluidic device was provided with a removable structure to separate two chambers, both containing the previously described aptamer-based biosensor and monocyte-like cells (U937 cell line) [261].

Due to such promising results obtained in 2D culture conditions, currently, there is a growing interest in adapting electrochemical aptasensors to 3D hydrogel-based cell culture systems. 

For instance, Su Ryon et al. designed a microfluidic electrochemical sensor functionalized with specific aptamers for the detection of cardiotoxic biomarkers and connected it to a custom-made perfusable bioreactor to investigate cardiac damages caused by cardiotoxic drugs. In particular, human embryonic stem cell-derived cardiomyocytes (hESC-CMs) spheroids were encapsulated within GelMA hydrogels and inserted within a bioreactor, where these were exposed to doxorubicin treatments. Levels of creatine kinase-MB, which is in vivo secreted from injured cardiac tissue, were measured, demonstrating significantly higher sensibility and stability of their probing system compared to Abs-based biosensors [262]. 

Likewise, drug-induced cardiotoxicity was studied in a very recent work, where a similar electrochemical apta-sensing platform was combined with a multi-organ-on-a-chip system interconnecting a heart-mimicking tissue and a breast cancer model. These tissues were modelled by embedding induced pluripotent stem cells (iPSCs) and breast cancer SK-BR-3 cells line spheroids in 3D GelMA hydrogels, respectively. In particular, troponin T, creatin kinase-MB and human epidermal growth factor receptor 2 (HER-2) secretion were monitored, upon doxorubicin administration, to investigate the relationship between chemotherapy-induced cardiotoxicity and breast cancer progression, both in physiological and pathological cardiac conditions. Optimal results were obtained both in terms of sensor performance compared with traditional ELISA assay, and in reproducing complex in vivo-like tissue interactions, since the cross-communication between health and cancer models affected the biomarkers production over time, significantly changing respect to the single organ models, when individually treated [263]. 

Nonetheless, although the organ-on-chip models allow working with low volumes and reproduction of fluid dynamic stimuli experienced from cells in the human body, they are characterized by too small matrices dimensions and cell numbers, which limit their translation to clinical/diagnostic applications due to the lack of physiologically-relevant sizes [58,166,264,265].

Moreover, it was demonstrated that the direct contact of aptamer-based electrochemical biosensors with biological complex culture media may affect sensors performance and stability due to the serum protein-dependent degradation of the sensing oligonucleotides; this, in turn, leads to the need for sample pretreatments or more complex chemical modification of the aptamers [239,266,267,268,269]. 

Therefore, to overcome these issues, novel culture model configurations and integration with aptasensors are required. To meet this need, Santos-Cancel et al. proposed to directly interface an electrochemical aptasensor with a 3D collagen I-based cell culture to realize a highly sensitive device, where gliotransmission mechanisms can be investigated in a brain-mimicking tissue. In this setting, the sensing element was placed within a commercial PMMA support and then an astrocytes-collagen mixed solution was poured over the biosensor, as illustrated in Figure 5d. Importantly, this PMMA holder was specifically adopted to work with larger matrix volumes, with respect to microfluidic-based cell cultures, to let cells grow in a more clinically-relevant size in vitro environment. In addition, by this approach, the collagen hydrogel prevented the sensing system from a harmful contact with culture medium components, acting as a protecting membrane without hampering the sensor function, as the same authors previously demonstrated [266,267]. This study aimed at continuously monitoring adenosine triphosphate (ATP) production, since it is well-known that astrocytes interactions occur via purinergic signaling through the secretion of gliotransmitters as ATP [270,271]. Long-lasting analyses were performed and high sensitivity to the continuous ATP changes was demonstrated. Interestingly, authors highlighted the potential of this platform to directly detect small molecule release in a complex in vivo-like surrounding environment with a high spatiotemporal resolution [272]. The biosensors mentioned have been resumed in Table 2.

## 5. Conclusions and Future Outlook

Biosensing tools able to perform automatic real-time readouts in 3D hydrogel-based in vitro models, as cutting-edge technologies, have been recently developed for several biomedical applications. Nevertheless, the advantages are still pretty limited, mainly due to the geometry of the hydrogels, which hampers the integration of sensor tools within 3D polymeric matrices without affecting their characteristics.

Although electrochemical and optical sensors allow the analyte to be probed with optimal sensitivity and stability, the impedance-based ones proved to be more suitable and versatile in many applications. 

In fact, these sensors provide a direct measure of cell viability, proliferation and aggregation in an easily integrable manner, without the use of additional invasive conjugating elements (e.g., micro- or nano-particles or genetically-encoded probes) which can impair the mechanical and biochemical features of the cells or scaffolds. In this respect, much more efforts remain to be generated to integrate a wider range of biosensors with 3D tissue-mimicking models to gain multiple information, without affecting the cell culture system integrity and stability. 

So far, researchers are moving towards the development of more clinically-relevant platforms where cells cultured within 3D systems can experience controlled fluidical and mechanical dynamic stimuli, as it happens in vivo. However, the integration of new-generation biosensors in 3D fluidic systems must be faced concurrently; indeed, microfluidic-based diagnostics can be foreseen as a key-driving feature also for an efficient and reliable point-of-care assessment of multiple targets, with reduced processing times and costs, and with a striking relevant social impact. Promising preliminary results have been obtained in evaluating cell signaling pathways by combining miniaturized electrochemical biosensors and micro-fluidic devices, but such applications are still at an early stage, due to the interference between the culture medium with the sensing element (i.e., Abs or aptamers), and also due to the fact that excessively small sensing volumes may affect proper capturing of the analyte molecule(s) of interest. Once these biases are overcome, however, the combination of engineered 3D cell systems cultured within fluidic platforms with automated, precise and real-time sensing tools, will remarkably favor a holistic assessment of cells condition, in highly reliable physio-pathological scenarios. Consequently, the scientific community will gain faster and high throughput data to properly feed, support and enhance in vitro-to-in vivo data extrapolation, both in the modeling and in the diagnostic field. 

## Figures and Tables

**Table 1:** Main advantages and disadvantages of the main pre-clinical tissue models.

Pre-Clinical Model	Main Advantages	Main Disadvantages
2D cell cultures	Simple to use	Limited or altered cell–cell and cell–extracellular matrix (ECM) interactions
	Cheap	Altered cell morphology, proliferation, and differentiation
	Standard	Overestimated drugs response
		Lack of metabolic gradients
		Oversimplified
Animal models	High complexity	Time-consuming, laborious, expensive
		Species-specific responses
		Ethical issues
3D cellular spheroids	Cheap	Lack of surrounding ECM
	Metabolic gradients	Susceptibility to physical deterioration
	Proper cell–cell interactions	
	In vivo-like cell morphology and proliferation	
3D hydrogel-based tissue models	Surrounding ECM with tunable properties	Batch-to-batch variability
	Reproduction of key mechanical and biochemical features of human tissues	Difficult to monitor cell activity with traditional tools
	Proper cell–cell and cell–ECM interactions	

**Table 2:** Summary of the biosensors analyzed in the current review with their main advantages and disadvantages.

	Biosensors to Monitor Cell Activity in 3D Hydrogel-Based Tissue Models			
Typology	Method or Technology of Detection	Advantages	Disadvantages	Ref
pH	Electrochemical	Fast and accurate	Require large sample volume and physical contact; provide only an average value	[85,86,87]
	ISFETs and LAPs	Sensitive and repetitive measurements; small sample volume	Require physical contact; limited applicability with 3D scaffolds	[64,88,89,90,91,92,93,94,95,96,97,98]
	Optical	Low costs; absence of immune and electrical interference; non-invasive sensing method; consistent, reliable, real-time, 3D measurements	Intrinsic cytotoxicity and photobleaching (fluorescent molecules); dye leaching; laborious (fluorophores with micro- or nano-particles)	[86,99,100,101,102,103,104,105,106,107,108,109,110,111,112]
Glucose	Electrochemical	High selectivity and sensitivity; low costs	Chemical and thermal instability of the enzymes, low versatility (enzyme-based); measure only culture medium concentration; limited lifetime, invasive, single-point measurement (microneedles)	[64,98,115,118,119,120,121,122,123,124,125,126,127]
	Optical	Real-time; 3D measurements	Limited applicability	[128,129,130]
Oxygen	Electrochemical	Real-time measurements; low costs	Susceptibility to contaminations (non-Clark-type); measure only culture medium concentration	[149,150,151,152,155]
	Optical	Effective; reliable; real-time; 3D measurements	Invasive and single-point measurement (optical fiber); provide only planar measurements (sensing film); laborious (fluorophores with micro- or nano-particles)	[136,156,157,158,159,160,161,162,163,164,165,169,170,171,172]
Impedance	-	Automation-compatible, label-free, real-time technology; versatility; non- invasive	May require multiparametric approach to for the interpretation of the data obtained (cause of impedance variations, such as hydrogel matrix degradation or cell migration, not detected)	[173,174,175,176,177,178,179,180,181,182,183,184,185,186,187,188,189,190]
Secreted molecules	Antibody-based	High specificity; quantification of molecules; small sample volume	Fast saturation limiting continuous analyses (need of reconfigurable systems or washing processes)	[205,222,225,226,227,232,237]
	Aptamer-based	Real-time; high specificity; quantification of molecules; small sample volume; high thermal and chemical stability; high sensibility; easily editable; simple conjugation with different labels	Sensibility to serum proteins of culture media (need of samples or aptamers pretreatments)	[258,260,261,262,263,272]

## Data Availability

Not applicable.

## References

[1] Hoarau-Véchot J., Rafii A., Touboul C., Pasquier J. (2018). Halfway between 2D and animal models: Are 3D cultures the ideal tool to study cancer-microenvironment interactions?. Int. J. Mol. Sci..

[2] Das V., Bruzzese F., Konečný P., Iannelli F., Budillon A., Hajdúch M. (2015). Pathophysiologically relevant in vitro tumor models for drug screening. Drug Discov. Today.

[3] Kapałczyńska M., Kolenda T., Przybyła W., Zajączkowska M., Teresiak A., Filas V., Ibbs M., Bliźniak R., Łuczewski Ł., Lamperska K. (2018). 2D and 3D cell cultures–a comparison of different types of cancer cell cultures. Arch. Med. Sci..

[4] Leong D.T., Ng K.W. (2014). Probing the relevance of 3D cancer models in nanomedicine research. Adv. Drug Deliv. Rev..

[5] Cukierman E., Pankov R., Stevens D.R., Yamada K.M. (2001). Taking cell-matrix adhesions to the third dimension. Science.

[6] MJ B., Aylin R., Saira I.M. (2003). Tissue architecture: The ultimate regulator of breast epithelial function. Curr. Opin. Cell Biol..

[7] Brien L.E.O., Zegers M.M.P., Mostov K.E. (2002). Culture Models. Group.

[8] Mulhall H.J., Hughes M.P., Kazmi B., Lewis M.P., Labeed F.H. (2013). Epithelial cancer cells exhibit different electrical properties when cultured in 2D and 3D environments. Biochim. Biophys. Acta–Gen. Subj..

[9] Tay C.Y., Pal M., Yu H., Leong W.S., Tan N.S., Ng K.W., Venkatraman S., Boey F., Leong D.T., Tan L.P. (2011). Bio-inspired micropatterned platform to steer stem cell differentiation. Small.

[10] Tay C.Y., Yu H., Pal M., Leong W.S., Tan N.S., Ng K.W., Leong D.T., Tan L.P. (2010). Micropatterned matrix directs differentiation of human mesenchymal stem cells towards myocardial lineage. Exp. Cell Res..

[11] Knight E., Przyborski S. (2015). Advances in 3D cell culture technologies enabling tissue-like structures to be created in vitro. J. Anat..

[12] Souza A.G., Ferreira I.C. (2016). Advances in Cell Culture: More than a Century after Cultivating Cells. J. Biotechnol. Biomater..

[13] Duval K., Grover H., Han L.H., Mou Y., Pegoraro A.F., Fredberg J., Chen Z. (2017). Modeling physiological events in 2D vs. 3D cell culture. Physiology.

[14] Xu X., Farach-Carson M.C., Jia X. (2014). Three-dimensional in vitro tumor models for cancer research and drug evaluation. Biotechnol. Adv..

[15] Fedi A., Vitale C., Ponschin G., Ayehunie S., Fato M., Scaglione S. (2021). In vitro models replicating the human intestinal epithelium for absorption and metabolism studies: A systematic review. J. Control. Release.

[16] Edmondson R., Broglie J.J., Adcock A.F., Yang L. (2014). Three-dimensional cell culture systems and their applications in drug discovery and cell-based biosensors. Assay Drug Dev. Technol..

[17] Marrella A., Dondero A., Aiello M., Casu B., Olive D., Regis S., Bottino C., Caluori G., Castriconi R., Scaglione S. (2019). Cell-laden hydrogel as a clinical-relevant 3D model for analyzing neuroblastoma growth, immunophenotype and susceptibility to therapies. Front. Immunol..

[18] Bhadriraju K., Chen C.S. (2002). Engineering cellular microenvironments to improve cell-based drug testing. Drug Discov. Today.

[19] Meigs L., Smirnova L., Rovida C., Leist M., Hartung T. (2018). Animal testing and its alternatives—The most important omics is economics. ALTEX.

[20] Romero L., Vela J.M. (2014). Alternative Models in Drug Discovery and Development Part I: In Silico and In Vitro Models. Vivo Model. Drug Discov..

[21] Cheluvappa R., Scowen P., Eri R. (2017). Ethics of animal research in human disease remediation, its institutional teaching; and alternatives to animal experimentation. Pharmacol. Res. Perspect..

[22] Liguori G.R., Jeronimus B.F., De Aquinas Liguori T.T., Moreira L.F.P., Harmsen M.C. (2017). Ethical Issues in the Use of Animal Models for Tissue Engineering: Reflections on Legal Aspects, Moral Theory, Three Rs Strategies, and Harm’Benefit Analysis. Tissue Eng. Part C Methods.

[23] Leist M., Hasiwa N., Rovida C., Daneshian M., Basketter D., Kimber I., Clewell H., Gocht T., Goldberg A., Busquet F. (2014). Consensus report on the future of animal-free systemic toxicity testing. ALTEX.

[24] Adler S., Basketter D., Creton S., Pelkonen O., Van Benthem J., Zuang V., Andersen K.E., Angers-Loustau A., Aptula A., Bal-Price A. (2011). Alternative (non-animal) methods for cosmetics testing: Current status and future prospects-2010. Arch. Toxicol..

[25] Hartung T., Blaauboer B.J., Bosgra S., Carney E., Coenen J., Conolly R.B., Corsini E., Green S., Faustman E.M., Gaspari A. (2011). T4 report. An expert consortium review of the EC-commissioned report “Alternative (non-animal) methods for cosmetics testing: Current status and future prospects-2010”. ALTEX.

[26] Dothel G., Vasina V., Barbara G., De Ponti F. (2013). Animal models of chemically induced intestinal inflammation: Predictivity and ethical issues. Pharmacol. Ther..

[27] Doke S.K., Dhawale S.C. (2015). Alternatives to animal testing: A review. Saudi Pharm. J..

[28] Baker B.M., Chen C.S. (2012). Deconstructing the third dimension-how 3D culture microenvironments alter cellular cues. J. Cell Sci..

[29] Horvath P., Aulner N., Bickle M., Davies A.M., Nery E.D., Ebner D., Montoya M.C., Östling P., Pietiäinen V., Price L.S. (2016). Screening out irrelevant cell-based models of disease. Nat. Rev. Drug Discov..

[30] Lancaster M.A., Renner M., Martin C.A., Wenzel D., Bicknell L.S., Hurles M.E., Homfray T., Penninger J.M., Jackson A.P., Knoblich J.A. (2013). Cerebral organoids model human brain development and microcephaly. Nature.

[31] Abbott A. (2003). Biology’s new dimension. Nature.

[32] Raghavan S., Ward M.R., Rowley K.R., Wold R.M., Takayama S., Buckanovich R.J., Mehta G. (2015). Formation of stable small cell number three-dimensional ovarian cancer spheroids using hanging drop arrays for preclinical drug sensitivity assays. Gynecol. Oncol..

[33] Herter S., Morra L., Schlenker R., Sulcova J., Fahrni L., Waldhauer I., Lehmann S., Reisländer T., Agarkova I., Kelm J.M. (2017). A novel three-dimensional heterotypic spheroid model for the assessment of the activity of cancer immunotherapy agents. Cancer Immunol. Immunother..

[34] Thoma C.R., Zimmermann M., Agarkova I., Kelm J.M., Krek W. (2014). 3D cell culture systems modeling tumor growth determinants in cancer target discovery. Adv. Drug Deliv. Rev..

[35] Longati P., Jia X., Eimer J., Wagman A., Witt M.R., Rehnmark S., Verbeke C., Toftgård R., Löhr M., Heuchel R.L. (2013). 3D pancreatic carcinoma spheroids induce a matrix-rich, chemoresistant phenotype offering a better model for drug testing. BMC Cancer.

[36] Costa E.C., Moreira A.F., de Melo-Diogo D., Gaspar V.M., Carvalho M.P., Correia I.J. (2016). 3D tumor spheroids: An overview on the tools and techniques used for their analysis. Biotechnol. Adv..

[37] Markovitz-Bishitz Y., Tauber Y., Afrimzon E., Zurgil N., Sobolev M., Shafran Y., Deutsch A., Howitz S., Deutsch M. (2010). A polymer microstructure array for the formation, culturing, and high throughput drug screening of breast cancer spheroids. Biomaterials.

[38] Asghar W., El Assal R., Shafiee H., Pitteri S., Paulmurugan R., Demirci U. (2015). Engineering cancer microenvironments for in vitro 3-D tumor models. Mater. Today.

[39] Infanger D.W., Lynch M.E., Fischbach C. (2013). Engineered Culture Models for Studies of Tumor- Microenvironment Interactions. Annu. Rev. Biomed. Eng..

[40] Alemany-ribes M., Semino C.E. (2014). Bioengineering 3D environments for cancer models. Adv. Drug Deliv. Rev..

[41] Gebeyehu A., Surapaneni S.K., Huang J., Mondal A., Wang V.Z., Haruna N.F., Bagde A., Arthur P., Kutlehria S., Patel N. (2021). Polysaccharide hydrogel based 3D printed tumor models for chemotherapeutic drug screening. Sci. Rep..

[42] Shi W., Huang J., Fang R., Liu M. (2020). Imparting functionality to the hydrogel by magnetic-field-induced nano-assembly and macro-response. ACS Appl. Mater. Interfaces.

[43] Hsieh F.-Y., Han H.-W., Chen X.-R., Yang C.-S., Wei Y., Hsu S. (2018). Non-viral delivery of an optogenetic tool into cells with self-healing hydrogel. Biomaterials.

[44] Young A.T., White O.C., Daniele M.A. (2020). Rheological properties of coordinated physical gelation and chemical crosslinking in gelatin methacryloyl (GelMA) hydrogels. Macromol. Biosci..

[45] Wang Y., Kankala R.K., Ou C., Chen A., Yang Z. (2022). Advances in hydrogel-based vascularized tissues for tissue repair and drug screening. Bioact. Mater..

[46] Alarçin E., Lee T.Y., Karuthedom S., Mohammadi M., Brennan M.A., Lee D.H., Marrella A., Zhang J., Syla D., Zhang Y.S. (2018). Injectable shear-thinning hydrogels for delivering osteogenic and angiogenic cells and growth factors. Biomater. Sci..

[47] Zhang J., Zheng Y., Lee J., Hua J., Li S., Panchamukhi A., Yue J., Gou X., Xia Z., Zhu L. (2021). A pulsatile release platform based on photo-induced imine-crosslinking hydrogel promotes scarless wound healing. Nat. Commun..

[48] Zhu D., Li Z., Huang K., Caranasos T.G., Rossi J.S., Cheng K. (2021). Minimally invasive delivery of therapeutic agents by hydrogel injection into the pericardial cavity for cardiac repair. Nat. Commun..

[49] Nicodemus G.D., Bryant S.J. (2008). Cell encapsulation in biodegradable hydrogels for tissue engineering applications. Tissue Eng. Part B Rev..

[50] Marrella A., Lagazzo A., Barberis F., Catelani T., Quarto R., Scaglione S. (2017). Enhanced mechanical performances and bioactivity of cell laden-graphene oxide/alginate hydrogels open new scenario for articular tissue engineering applications. Carbon.

[51] Tibbitt M.W., Anseth K.S. (2009). Hydrogels as extracellular matrix mimics for 3D cell culture. Biotechnol. Bioeng..

[52] Unal A.Z., West J.L. (2020). Synthetic ECM: Bioactive synthetic hydrogels for 3D tissue engineering. Bioconjug. Chem..

[53] Lee S.H., Shim K.Y., Kim B., Sung J.H. (2017). Hydrogel-based three-dimensional cell culture for organ-on-a-chip applications. Biotechnol. Prog..

[54] Marrella A., Lagazzo A., Dellacasa E., Pasquini C., Finocchio E., Barberis F., Pastorino L., Giannoni P., Scaglione S. (2018). 3D porous gelatin/PVA hydrogel as meniscus substitute using alginate micro-particles as porogens. Polymers.

[55] Song R., Murphy M., Li C., Ting K., Soo C., Zheng Z. (2018). Current development of biodegradable polymeric materials for biomedical applications. Drug Des. Dev. Ther..

[56] Schnabel-Lubovsky M., Kossover O., Melino S., Nanni F., Talmon Y., Seliktar D. (2019). Visualizing cell-laden fibrin-based hydrogels using cryogenic scanning electron microscopy and confocal microscopy. J. Tissue Eng. Regen. Med..

[57] Sohn H.W., Tolar P., Brzostowski J., Pierce S.K. (2010). Imaging and analysis of three-dimensional cell culture models. Methods Mol. Biol..

[58] Marrella A. (2020). 3D fluid-dynamic ovarian cancer model resembling systemic drug administration for efficacy assay. ALTEX.

[59] Hasan A., Nurunnabi M., Morshed M., Paul A., Polini A., Kuila T., Al Hariri M., Lee Y., Jaffa A.A. (2014). Recent advances in application of biosensors in tissue engineering. Biomed Res. Int..

[60] Li Y.-C.E., Lee I. (2020). The current trends of biosensors in tissue engineering. Biosensors.

[61] Purohit B., Vernekar P.R., Shetti N.P., Chandra P. (2020). Biosensor nanoengineering: Design, operation, and implementation for biomolecular analysis. Sensors Int..

[62] Kratz S.R.A., Höll G., Schuller P., Ertl P., Rothbauer M. (2019). Latest trends in biosensing for microphysiological organs-on-a-chip and body-on-a-chip systems. Biosensors.

[63] Caballero D., Kaushik S., Correlo V.M., Oliveira J.M., Reis R.L., Kundu S.C. (2017). Organ-on-chip models of cancer metastasis for future personalized medicine: From chip to the patient. Biomaterials.

[64] Kieninger J., Weltin A., Flamm H., Urban G.A. (2018). Microsensor systems for cell metabolism-from 2D culture to organ-on-chip. Lab Chip.

[65] Bousse L. (1996). Whole cell biosensors. Sens. Actuators B Chem..

[66] Du H., Strohsahl C.M., Camera J., Miller B.L., Krauss T.D. (2005). Sensitivity and specificity of metal surface-immobilized “molecular beacon” biosensors. J. Am. Chem. Soc..

[67] Mehrotra P. (2016). Biosensors and their applications A review. J. Oral Biol. Craniofacial Res..

[68] Alhadrami H.A. (2018). Biosensors: Classifications, medical applications, and future prospective. Biotechnol. Appl. Biochem..

[69] Hu N., Ha D., Wu C., Zhou J., Kirsanov D., Legin A., Wang P. (2012). A LAPS array with low cross-talk for non-invasive measurement of cellular metabolism. Sens. Actuators A Phys..

[70] Mandel L.J., Kleinzeller A.B.T.-C.T. (1986). Chapter 8 Energy Metabolism of Cellular Activation, Growth, and Transformation. The Role of Membranes in Cell Growth and Differentiation.

[71] Owicki J.C., Wallace Parce J. (1992). Biosensors based on the energy metabolism of living cells: The physical chemistry and cell biology of extracellular acidification. Biosens. Bioelectron..

[72] Lehmann M., Baumann W., Brischwein M., Ehret R., Kraus M., Schwinde A., Bitzenhofer M., Freund I., Wolf B. (2000). Non-invasive measurement of cell membrane associated proton gradients by ion-sensitive field effect transistor arrays for microphysiological and bioelectronical applications. Biosens. Bioelectron..

[73] Wolf B., Brischwein M., Baumann W., Ehret R., Kraus M. (1998). Monitoring of cellular signalling and metabolism with modular sensor- technique: The physiocontrol-microsystem (PCM®). Biosens. Bioelectron..

[74] Zhang X., Lin Y., Gillies R.J. (2010). Tumor pH and its measurement. J. Nucl. Med..

[75] Stock C., Schwab A. (2009). Protons make tumor cells move like clockwork. Pflug. Arch. Eur. J. Physiol..

[76] Singh K., Ohlan A., Saini P., Dhawan S.K. (2008). pH sensor based on polyaniline and aniline–anthranilic acid copolymer films using quartz crystal microbalance and electronic absorption spectroscopy. Polym. Adv. Technol..

[77] Martin N.K., Gaffney E.A., Gatenby R.A., Maini P.K. (2010). Tumour-stromal interactions in acid-mediated invasion: A mathematical model. J. Theor. Biol..

[78] Chu B., Wang H., Song B., Peng F., Su Y., He Y. (2016). Fluorescent and Photostable Silicon Nanoparticles Sensors for Real-Time and Long-Term Intracellular pH Measurement in Live Cells. Anal. Chem..

[79] Wike-Hooley J.L., van den Berg A.P., van der Zee J., Reinhold H.S. (1985). Human tumour pH and its variation. Eur. J. Cancer Clin. Oncol..

[80] Ges I.A., Ivanov B.L., Schaffer D.K., Lima E.A., Werdich A.A., Baudenbacher F.J. (2005). Thin-film IrOx pH microelectrode for microfluidic-based microsystems. Biosens. Bioelectron..

[81] Lin C.F., Lee G.B., Wang C.H., Lee H.H., Liao W.Y., Chou T.C. (2006). Microfluidic pH-sensing chips integrated with pneumatic fluid-control devices. Biosens. Bioelectron..

[82] Milgrew M.J., Riehle M.O., Cumming D.R.S. (2005). A large transistor-based sensor array chip for direct extracellular imaging. Sens. Actuators B Chem..

[83] Yadavalli V.K., Pishko M.V. (2004). Biosensing in microfluidic channels using fluorescence polarization. Anal. Chim. Acta.

[84] Liu Z., Liu J., Chen T. (2005). Phenol red immobilized PVA membrane for an optical pH sensor with two determination ranges and long-term stability. Sens. Actuators B Chem..

[85] Park C.H., Lee S., Pornnoppadol G., Nam Y.S., Kim S.H., Kim B.J. (2018). Microcapsules Containing pH-Responsive, Fluorescent Polymer-Integrated MoS2: An Effective Platform for in Situ pH Sensing and Photothermal Heating. ACS Appl. Mater. Interfaces.

[86] Kattipparambil Rajan D., Patrikoski M., Verho J., Sivula J., Ihalainen H., Miettinen S., Lekkala J. (2016). Optical non-contact pH measurement in cell culture with sterilizable, modular parts. Talanta.

[87] Moldero I.L., Chandra A., Cavo M., Mota C., Kapsokalyvas D., Gigli G., Moroni L., del Mercato L.L. (2020). Probing the pH Microenvironment of Mesenchymal Stromal Cell Cultures on Additive-Manufactured Scaffolds. Small.

[88] Lu C., Hou T., Pan T. (2018). High-Performance Double-Gate. IEEE Trans. Electron Devices.

[89] Lai C.S., Yang C.M., Lu T.F. (2006). PH sensitivity improvement on 8 nm thick hafnium oxide by post deposition annealing. Electrochem. Solid-State Lett..

[90] Pan T.M., Liao K.M. (2007). Structural properties and sensing characteristics of Y2O3 sensing membrane for pH-ISFET. Sens. Actuators B Chem..

[91] Rani R.A., Sidek O. ISFET pH sensor characterization: Towards biosensor microchip application. Proceedings of the 2004 IEEE Region 10 Conference TENCON 2004.

[92] Lehmann M., Baumann W., Brischwein M., Gahle H.J., Freund I., Ehret R., Drechsler S., Palzer H., Kleintges M., Sieben U. (2001). Simultaneous measurement of cellular respiration and acidification with a single CMOS ISFET. Biosens. Bioelectron..

[93] Owicki J.C., Bousse L.J., Hafeman D.G., Kirk G.L., Olson J.D., Wada H.G., Parce J.W. (1994). The Light-Addressable Potentiometric Sensor: Principles and Biological Applications. Annu. Rev. Biophys. Biomol. Struct..

[94] Yoshinobu T., Schöning M.J. (2021). Light-addressable potentiometric sensors (LAPS) for cell monitoring and biosensing. Curr. Opin. Electrochem..

[95] Hu N., Wu C., Ha D., Wang T., Liu Q., Wang P. (2013). A novel microphysiometer based on high sensitivity LAPS and microfluidic system for cellular metabolism study and rapid drug screening. Biosens. Bioelectron..

[96] Shaibani P.M., Etayash H., Naicker S., Kaur K., Thundat T. (2017). Metabolic Study of Cancer Cells Using a pH Sensitive Hydrogel Nanofiber Light Addressable Potentiometric Sensor. ACS Sens..

[97] Yang C.-M., Yen T., Liu H.-L., Lin Y.-J., Lin P.-Y., Tsui L.S., Chen C.-H., Chen Y.-P., Hsu Y.-C., Lo C.-H. (2021). A real-time mirror-LAPS mini system for dynamic chemical imaging and cell acidification monitoring. Sens. Actuators B Chem..

[98] Zhang H.W., Hu X.B., Qin Y., Jin Z.H., Zhang X.W., Liu Y.L., Huang W.H. (2019). Conductive Polymer Coated Scaffold to Integrate 3D Cell Culture with Electrochemical Sensing. Anal. Chem..

[99] Wu M.H., Lin J.L., Wang J., Cui Z., Cui Z. (2009). Development of high throughput optical sensor array for on-line pH monitoring in micro-scale cell culture environment. Biomed. Microdevices.

[100] Wikswo J.P., Block F.E., Cliffel D.E., Goodwin C.R., Marasco C.C., Markov D.A., McLean D.L., McLean J.A., McKenzie J.R., Reiserer R.S. (2013). Engineering challenges for instrumenting and controlling integrated organ-on-chip systems. IEEE Trans. Biomed. Eng..

[101] Shaegh S.A.M., De Ferrari F., Zhang Y.S., Nabavinia M., Mohammad N.B., Ryan J., Pourmand A., Laukaitis E., Sadeghian R.B., Nadhman A. (2016). A microfluidic optical platform for real-time monitoring of pH and oxygen in microfluidic bioreactors and organ-on-chip devices. Biomicrofluidics.

[102] Wu S., Wu S., Yi Z., Zeng F., Wu W., Qiao Y., Zhao X., Cheng X., Tian Y. (2018). Hydrogel-based fluorescent dual pH and oxygen sensors loaded in 96-well plates for high-throughput cell metabolism studies. Sensors.

[103] Hanson M.A., Ge X., Kostov Y., Brorson K.A., Moreira A.R., Rao G. (2007). Comparisons of optical pH and dissolved oxygen sensors with traditional electrochemical probes during mammalian cell culture. Biotechnol. Bioeng..

[104] Naciri M., Kuystermans D., Al-Rubeai M. (2008). Monitoring pH and dissolved oxygen in mammalian cell culture using optical sensors. Cytotechnology.

[105] Arik M., Çelebi N., Onganer Y. (2005). Fluorescence quenching of fluorescein with molecular oxygen in solution. J. Photochem. Photobiol. A Chem..

[106] Buckler K.J., Vaughan-Jones R.D. (1990). Application of a new pH-sensitive fluoroprobe (carboxy-SNARF-1) for intracellular pH measurement in small, isolated cells. Pflügers Arch. Eur. J. Physiol..

[107] Kenney R.M., Boyce M.W., Whitman N.A., Kromhout B.P., Lockett M.R. (2018). A pH-Sensing Optode for Mapping Spatiotemporal Gradients in 3D Paper-Based Cell Cultures. Anal. Chem..

[108] Chandra A., Singh N. (2017). Cell Microenvironment pH Sensing in 3D Microgels Using Fluorescent Carbon Dots. ACS Biomater. Sci. Eng..

[109] Shangguan J., He D., He X., Wang K., Xu F., Liu J., Tang J., Yang X., Huang J. (2016). Label-Free Carbon-Dots-Based Ratiometric Fluorescence pH Nanoprobes for Intracellular pH Sensing. Anal. Chem..

[110] Wang C., Xu Z., Zhang C. (2015). Polyethyleneimine-Functionalized Fluorescent Carbon Dots: Water Stability, pH Sensing, and Cellular Imaging. ChemNanoMat.

[111] Du F., Ming Y., Zeng F., Yu C., Wu S. (2013). A low cytotoxic and ratiometric fluorescent nanosensor based on carbon-dots for intracellular pH sensing and mapping. Nanotechnology.

[112] Ke G., Zhu Z., Wang W., Zou Y., Guan Z., Jia S., Zhang H., Wu X., Yang C.J. (2014). A cell-surface-anchored ratiometric fluorescent probe for extracellular pH sensing. ACS Appl. Mater. Interfaces.

[113] Fan Y., Jimenez Del Val I., Müller C., Wagtberg Sen J., Rasmussen S.K., Kontoravdi C., Weilguny D., Andersen M.R. (2015). Amino acid and glucose metabolism in fed-batch CHO cell culture affects antibody production and glycosylation. Biotechnol. Bioeng..

[114] Quek L.-E., Dietmair S., Krömer J.O., Nielsen L.K. (2010). Metabolic flux analysis in mammalian cell culture. Metab. Eng..

[115] Boero C., Carrara S., Del Vecchio G., Calzà L., De Micheli G. (2011). Highly sensitive carbon nanotube-based sensing for lactate and glucose monitoring in cell culture. IEEE Trans. Nanobiosci..

[116] Borgmann S., Schulte A., Neugebauer S., Schuhmann W. (2012). Amperometric Biosensors.

[117] Heller A., Feldman B. (2008). Electrochemical Glucose Sensors and Their Applications in Diabetes Management. Chem. Rev..

[118] Obregón R., Ahadian S., Ramón-Azcón J., Chen L., Fujita T., Shiku H., Chen M., Matsue T. (2013). Non-invasive measurement of glucose uptake of skeletal muscle tissue models using a glucose nanobiosensor. Biosens. Bioelectron..

[119] Chang D.K., Goel A., Ricciardiello L., Lee D.H., Chang C.L., Carethers J.M., Boland C.R. (2003). Effect of H2O2 on cell cycle and survival in DNA mismatch repair-deficient and -proficient cell lines. Cancer Lett..

[120] Pereira Rodrigues N., Sakai Y., Fujii T. (2008). Cell-based microfluidic biochip for the electrochemical real-time monitoring of glucose and oxygen. Sens. Actuators B Chem..

[121] Weltin A., Slotwinski K., Kieninger J., Moser I., Jobst G., Wego M., Ehret R., Urban G.A. (2014). Cell culture monitoring for drug screening and cancer research: A transparent, microfluidic, multi-sensor microsystem. Lab Chip.

[122] Kemas A.M., Youhanna S., Zandi Shafagh R., Lauschke V.M. (2021). Insulin-dependent glucose consumption dynamics in 3D primary human liver cultures measured by a sensitive and specific glucose sensor with nanoliter input volume. FASEB J..

[123] Ma Z., Luo Y., Zhu Q., Jiang M., Pan M., Xie T., Huang X., Chen D. (2020). In-situ monitoring of glucose metabolism in cancer cell microenvironments based on hollow fiber structure. Biosens. Bioelectron..

[124] Bavli D., Prill S., Ezra E., Levy G., Cohen M., Vinken M., Vanfleteren J., Jaeger M., Nahmias Y. (2016). Real-time monitoring of metabolic function in liver-onchip microdevices tracks the dynamics of Mitochondrial dysfunction. Proc. Natl. Acad. Sci. USA..

[125] Qian L.H., Chen M.W. (2007). Ultrafine nanoporous gold by low-temperature dealloying and kinetics of nanopore formation. Appl. Phys. Lett..

[126] Figueiredo L., Pace R., D’Arros C., Réthoré G., Guicheux J., Le Visage C., Weiss P. (2018). Assessing glucose and oxygen diffusion in hydrogels for the rational design of 3D stem cell scaffolds in regenerative medicine. J. Tissue Eng. Regen. Med..

[127] Farrell M.J., Shin J.I., Smith L.J., Mauck R.L. (2015). Functional consequences of glucose and oxygen deprivation onengineered mesenchymal stem cell-based cartilage constructs. Osteoarthr. Cartil..

[128] Li I.T., Pham E., Truong K. (2006). Protein biosensors based on the principle of fluorescence resonance energy transfer for monitoring cellular dynamics. Biotechnol. Lett..

[129] Lorenzo C., Frongia C., Jorand R., Fehrenbach J., Weiss P., Maandhui A., Gay G., Ducommun B., Lobjois V. (2011). Live cell division dynamics monitoring in 3D large spheroid tumor models using light sheet microscopy. Cell Div..

[130] Maioli V., Chennell G., Sparks H., Lana T., Kumar S., Carling D., Sardini A., Dunsby C. (2016). Time-lapse 3-D measurements of a glucose biosensor in multicellular spheroids by light sheet fluorescence microscopy in commercial 96-well plates. Sci. Rep..

[131] Lewis D.M., Blatchley M.R., Park K.M., Gerecht S. (2017). O2-controllable hydrogels for studying cellular responses to hypoxic gradients in three dimensions in vitro and in vivo. Nat. Protoc..

[132] Schmitz C., Pepelanova I., Seliktar D., Potekhina E., Belousov V.V., Scheper T., Lavrentieva A. (2020). Live reporting for hypoxia: Hypoxia sensor–modified mesenchymal stem cells as in vitro reporters. Biotechnol. Bioeng..

[133] Rouwkema J., Koopman B.F.J.M., Blitterswijk C.A.V., Dhert W.J.A., Malda J. (2009). Supply of nutrients to cells in engineered tissues. Biotechnol. Genet. Eng. Rev..

[134] (2003). Christopher W Pugh; Peter J Ratcliffe Regulation of angiogenesis by hypoxia: Role of the HIF system. Nat. Med..

[135] Magliaro C., Mattei G., Iacoangeli F., Corti A., Piemonte V., Ahluwalia A., Lewis D.M., Park K.M., Tang V., Xu Y. (2016). Hypoxia in Static and Dynamic 3D Culture Systems for Tissue Engineering of Bone. Free Radic. Biol. Med..

[136] Acosta M.A., Ymele-Leki P., Kostov Y.V., Leach J.B. (2009). Fluorescent microparticles for sensing cell microenvironment oxygen levels within 3D scaffolds. Biomaterials.

[137] Westfall S.D., Sachdev S., Das P., Hearne L.B., Hannink M., Roberts R.M., Ezashi T. (2008). Identification of oxygen-sensitive transcriptional programs in human embryonic stem cells. Stem Cells Dev..

[138] O’Driscoll S.W., Fitzsimmons J.S., Commisso C.N. (1997). Role of oxygen tension during cartilage formation by periosteum. J. Orthop. Res..

[139] Mohyeldin A., Garzón-Muvdi T., Quiñones-Hinojosa A. (2010). Oxygen in stem cell biology: A critical component of the stem cell niche. Cell Stem Cell.

[140] Zhang K., Zhu L., Fan M. (2011). Oxygen, a Key Factor Regulating Cell Behavior during Neurogenesis and Cerebral Diseases. Front. Mol. Neurosci..

[141] Carreau A., Hafny-Rahbi B.E., Matejuk A., Grillon C., Kieda C. (2011). Why is the partial oxygen pressure of human tissues a crucial parameter? Small molecules and hypoxia. J. Cell. Mol. Med..

[142] Pouysségur J., Dayan F., Mazure N.M. (2006). Hypoxia signalling in cancer and approaches to enforce tumour regression. Nature.

[143] Harrison B.S., Eberli D., Lee S.J., Atala A., Yoo J.J. (2007). Oxygen producing biomaterials for tissue regeneration. Biomaterials.

[144] Hossmann K.-A. (2006). Pathophysiology and Therapy of Experimental Stroke. Cell. Mol. Neurobiol..

[145] Heathman T.R.J., Nienow A.W., McCall M.J., Coopman K., Kara B., Hewitt C.J. (2015). The translation of cell-based therapies: Clinical landscape and manufacturing challenges. Regen. Med..

[146] Beckers S., Noor F., Müller-Vieira U., Mayer M., Strigun A., Heinzle E. (2010). High throughput, non-invasive and dynamic toxicity screening on adherent cells using respiratory measurements. Toxicol. In Vitro.

[147] Wouters A., Pauwels B., Lambrechts H.A.J., Pattyn G.G.O., Ides J., Baay M., Meijnders P., Dewilde S., Vermorken J.B., Lardon F. (2009). Chemoradiation interactions under reduced oxygen conditions: Cellular characteristics of an in vitro model. Cancer Lett..

[148] Browne S.M., Daud H., Murphy W.G., Al-Rubeai M. (2014). Measuring dissolved oxygen to track erythroid differentiation of hematopoietic progenitor cells in culture. J. Biotechnol..

[149] Wu C.C., Luk H.N., Lin Y.T.T., Yuan C.Y. (2010). A Clark-type oxygen chip for in situ estimation of the respiratory activity of adhering cells. Talanta.

[150] Kieninger J., Aravindalochanan K., Sandvik J.A., Pettersen E.O., Urban G.A. (2014). Pericellular oxygen monitoring with integrated sensor chips for reproducible cell culture experiments. Cell Prolif..

[151] Liebisch F., Weltin A., Marzioch J., Urban G.A., Kieninger J. (2020). Zero-consumption Clark-type microsensor for oxygen monitoring in cell culture and organ-on-chip systems. Sens. Actuators B Chem..

[152] Kieninger J., Tamari Y., Enderle B., Jobst G., Sandvik J.A., Pettersen E.O., Urban G.A. (2018). Sensor access to the cellular microenvironment using the sensing cell culture flask. Biosensors.

[153] Wilson R.L., Connell J.P., Grande-Allen K.J. (2019). Monitoring Oxygen Levels within Large, Tissue-Engineered Constructs Using Porphyin-Hydrogel Microparticles. ACS Biomater. Sci. Eng..

[154] Magliaro C., Mattei G., Iacoangeli F., Corti A., Piemonte V., Ahluwalia A. (2019). Oxygen consumption characteristics in 3D constructs depend on cell density. Front. Bioeng. Biotechnol..

[155] Weltin A., Hammer S., Noor F., Kaminski Y., Kieninger J., Urban G.A. (2017). Accessing 3D microtissue metabolism: Lactate and oxygen monitoring in hepatocyte spheroids. Biosens. Bioelectron..

[156] Figueiredo L., Le Visage C., Weiss P., Yang J. (2020). Quantifying oxygen levels in 3D bioprinted cell-laden thick constructs with perfusable microchannel networks. Polymers.

[157] Marland J.R.K., Gray M.E., Dunare C., Blair E.O., Tsiamis A., Sullivan P., González-Fernández E., Greenhalgh S.N., Gregson R., Clutton R.E. (2020). Real-time measurement of tumour hypoxia using an implantable microfabricated oxygen sensor. Sens. Bio-Sens. Res..

[158] Wolfbeis O.S. (2015). Luminescent sensing and imaging of oxygen: Fierce competition to the Clark electrode. BioEssays.

[159] Otero J., Ulldemolins A., Farré R., Almendros I. (2021). Oxygen Biosensors and Control in 3D Physiomimetic Experimental Models. Antioxidants.

[160] Rivera K.R., Pozdin V.A., Young A.T., Erb P.D., Wisniewski N.A., Magness S.T., Daniele M. (2019). Integrated phosphorescence-based photonic biosensor (iPOB) for monitoring oxygen levels in 3D cell culture systems. Biosens. Bioelectron..

[161] Boyce M.W., Kenney R.M., Truong A.S., Lockett M.R. (2016). Quantifying oxygen in paper-based cell cultures with luminescent thin film sensors Young Investigators in Analytical and Bioanalytical Science. Anal. Bioanal. Chem..

[162] Boyce M.W., Simke W.C., Kenney R.M., Lockett M.R. (2019). Generating linear oxygen gradients across 3D cell cultures with block-layered oxygen controlled chips (BLOCCs). Anal. Methods.

[163] Wolff P., Heimann L., Liebsch G., Meier R.J., Gutbrod M., van Griensven M., Balmayor E.R. (2019). Oxygen-distribution within 3-D collagen I hydrogels for bone tissue engineering. Mater. Sci. Eng. C.

[164] Li K., Liu B., Biosciences M., Biosciences M., Papkovsky D.B., Zhdanov A.V. (2016). Polymer-encapsulated organic nanoparticles for fluorescence and photoacoustic imaging. Free Radic. Biol. Med..

[165] Koduri M.P., Goudar V.S., Shao Y.W., Hunt J.A., Henstock J.R., Curran J., Tseng F.G. (2018). Fluorescence-Based Nano-Oxygen Particles for Spatiometric Monitoring of Cell Physiological Conditions. ACS Appl. Mater. Interfaces.

[166] Vitale C., Fedi A., Marrella A., Varani G., Fato M., Scaglione S. (2020). 3D perfusable hydrogel recapitulating the cancer dynamic environment to in vitro investigate metastatic colonization. Polymers.

[167] Id A.M., Fedi A., Varani G., Vaccari I., Id M.F., Firpo G., Guida P., Aceto N., Id S.S. (2021). High blood flow shear stress values are associated with circulating tumor cells cluster disaggregation in a multi-channel microfluidic device. PLoS ONE.

[168] Pulsoni I., Lubda M., Aiello M., Fedi A., Marzagalli M., von Hagen J., Scaglione S. (2022). Comparison Between Franz Diffusion Cell and a novel Micro-physiological System for In Vitro Penetration Assay Using Different Skin Models. SLAS Technol..

[169] Lesher-Pérez S.C., Kim G.A., Kuo C.H., Leung B.M., Mong S., Kojima T., Moraes C., Thouless M.D., Luker G.D., Takayama S. (2017). Dispersible oxygen microsensors map oxygen gradients in three-dimensional cell cultures. Biomater. Sci..

[170] Dmitriev R.I., Papkovsky D.B. (2012). Optical probes and techniques for O2 measurement in live cells and tissue. Cell. Mol. Life Sci..

[171] Leedale J., Herrmann A., Bagnall J., Fercher A., Papkovsky D., Sée V., Bearon R.N. (2014). Modeling the dynamics of hypoxia inducible factor-1α (HIF-1α) within single cells and 3D cell culture systems. Math. Biosci..

[172] Papkovsky D.B., Zhdanov A.V. (2016). Phosphorescence based O2 sensors Essential tools for monitoring cell and tissue oxygenation and its impact on metabolism. Free Radic. Biol. Med..

[173] Asphahani F., Zhang M. (2007). Cellular impedance biosensors for drug screening and toxin detection. Analyst.

[174] Hu N., Wang T., Wang Q., Zhou J., Zou L., Su K., Wu J., Wang P. (2015). High-performance beating pattern function of human induced pluripotent stem cell-derived cardiomyocyte-based biosensors for hERG inhibition recognition. Biosens. Bioelectron..

[175] Liu Q., Wu C., Cai H., Hu N., Zhou J., Wang P. (2014). Cell-Based Biosensors and Their Application in Biomedicine. Chem. Rev..

[176] Nguyen T.A., Yin T.-I., Reyes D., Urban G.A. (2013). Microfluidic Chip with Integrated Electrical Cell-Impedance Sensing for Monitoring Single Cancer Cell Migration in Three-Dimensional Matrixes. Anal. Chem..

[177] Zhou J., Wu C., Tu J., Ling Y., Hu N., Zhang Y., Su K., Wang P. (2013). Assessment of cadmium-induced hepatotoxicity and protective effects of zinc against it using an improved cell-based biosensor. Sens. Actuators A Phys..

[178] Zou L., Wu C., Wang Q., Zhou J., Su K., Li H., Hu N., Wang P. (2015). An improved sensitive assay for the detection of PSP toxins with neuroblastoma cell-based impedance biosensor. Biosens. Bioelectron..

[179] Heileman K., Daoud J., Tabrizian M. (2013). Dielectric spectroscopy as a viable biosensing tool for cell and tissue characterization and analysis. Biosens. Bioelectron..

[180] Montaño-Figueroa A.G., Wheelis S.E., Hedden B.M., Alshareef N.H., Dammanna D., Shaik H., Rodrigues D.C., Quevedo-Lopez M. (2019). Detection of apoptotic and live pre-osteoblast cell line using impedance-based biosensors with variable electrode design. Biosens. Bioelectron..

[181] Xia N., Huang Y., Cui Z., Liu S., Deng D., Liu L., Wang J. (2020). Impedimetric biosensor for assay of caspase-3 activity and evaluation of cell apoptosis using self-assembled biotin-phenylalanine network as signal enhancer. Sens. Actuators B Chem..

[182] Anh-Nguyen T., Tiberius B., Pliquett U., Urban G.A. (2016). An impedance biosensor for monitoring cancer cell attachment, spreading and drug-induced apoptosis. Sens. Actuators A Phys..

[183] Benson K., Cramer S., Galla H.-J. (2013). Impedance-based cell monitoring: Barrier properties and beyond. Fluids Barriers CNS.

[184] Elbrecht D.H., Long C.J., Hickman J.J. (2016). Transepithelial/endothelial Electrical Resistance (TEER) theory and ap-plications for microfluidic body-on-a-chip devices. J. Rare Dis. Res. Treat..

[185] Srinivasan B., Kolli A.R., Esch M.B., Abaci H.E., Shuler M.L., Hickman J.J. (2015). TEER Measurement Techniques for In Vitro Barrier Model Systems. J. Lab. Autom..

[186] Thakur M., Mergel K., Weng A., Frech S., Gilabert-Oriol R., Bachran D., Melzig M.F., Fuchs H. (2012). Real time monitoring of the cell viability during treatment with tumor-targeted toxins and saponins using impedance measurement. Biosens. Bioelectron..

[187] Canali C., Heiskanen A., Martinsen Ø.G., Mohanty S., Dufva M., Wolff A., Emnéus J. (2016). Impedance-based monitoring for tissue engineering applications. Proceedings of the II Latin American Conference on Bioimpedance.

[188] Pan Y., Hu N., Wei X., Gong L., Zhang B., Wan H., Wang P. (2019). 3D cell-based biosensor for cell viability and drug assessment by 3D electric cell/matrigel-substrate impedance sensing. Biosens. Bioelectron..

[189] Lei K.F., Liu T.K., Tsang N.M. (2018). Towards a high throughput impedimetric screening of chemosensitivity of cancer cells suspended in hydrogel and cultured in a paper substrate. Biosens. Bioelectron..

[190] Lei K.F., Wu Z.M., Huang C.H. (2015). Impedimetric quantification of the formation process and the chemosensitivity of cancer cell colonies suspended in 3D environment. Biosens. Bioelectron..

[191] Jeddi I., Revzin A. (2012). Sensing cell-secreted molecules. Bioanal. Rev..

[192] Maruvada P., Wang W., Wagner P.D., Srivastava S. (2005). Biomarkers in molecular medicine: Cancer detection and diagnosis. Biotechniques.

[193] Gnecchi M., Zhang Z., Ni A., Dzau V.J. (2008). Paracrine mechanisms in adult stem cell signaling and therapy. Circ. Res..

[194] Lander A.D. (2013). How cells know where they are. Science.

[195] Stastna M., Van Eyk J.E. (2012). Secreted proteins as a fundamental source for biomarker discovery. Proteomics.

[196] Borish L.C., Steinke J.W. (2003). 2. Cytokines and chemokines. J. Allergy Clin. Immunol..

[197] Turner M.D., Nedjai B., Hurst T., Pennington D.J. (2014). Cytokines and chemokines: At the crossroads of cell signalling and inflammatory disease. Biochim. Biophys. Acta Mol. Cell Res..

[198] Rothenberg E.V. (2007). Cell lineage regulators in B and T cell development. Nat. Immunol..

[199] Brando B., Barnett D., Janossy G., Mandy F., Autran B., Rothe G., Scarpati B., D’Avanzo G., D’Hautcourt J.L., Lenkei R. (2000). Cytofluorometric methods for assessing absolute numbers of cell subsets in blood. Commun. Clin. Cytom..

[200] Cox J.H., Ferrari G., Janetzki S. (2006). Measurement of cytokine release at the single cell level using the ELISPOT assay. Methods.

[201] Karlsson A.C., Martin J.N., Younger S.R., Bredt B.M., Epling L., Ronquillo R., Varma A., Deeks S.G., McCune J.M., Nixon D.F. (2003). Comparison of the ELISPOT and cytokine flow cytometry assays for the enumeration of antigen-specific T cells. J. Immunol. Methods.

[202] Mäkitalo B., Andersson M., Areström I., Karlén K., Villinger F., Ansari A., Paulie S., Thorstensson R., Ahlborg N. (2002). ELISpot and ELISA analysis of spontaneous, mitogen-induced and antigen-specific cytokine production in cynomolgus and rhesus macaques. J. Immunol. Methods.

[203] Steffen M.J., Ebersole J.L. (1996). Sequential ELISA for cytokine levels in limited volumes of biological fluids. Biotechniques.

[204] Son K.J., Rahimian A., Shin D.S., Siltanen C., Patel T., Revzin A. (2016). Microfluidic compartments with sensing microbeads for dynamic monitoring of cytokine and exosome release from single cells. Analyst.

[205] Shin S.R., Kilic T., Zhang Y.S., Avci H., Hu N., Kim D., Branco C., Aleman J., Massa S., Silvestri A. (2017). Label-Free and Regenerative Electrochemical Microfluidic Biosensors for Continual Monitoring of Cell Secretomes. Adv. Sci..

[206] Cappi G., Spiga F.M., Moncada Y., Ferretti A., Beyeler M., Bianchessi M., Decosterd L., Buclin T., Guiducci C. (2015). Label-Free detection of tobramycin in serum by transmission-localized surface plasmon resonance. Anal. Chem..

[207] Fortier M.H., Bonneil E., Goodley P., Thibault P. (2005). Integrated microfluidic device for mass spectrometry-based proteomics and its application to biomarker discovery programs. Anal. Chem..

[208] Sivanesan A., Izake E.L., Agoston R., Ayoko G.A., Sillence M. (2015). Reproducible and label-free biosensor for the selective extraction and rapid detection of proteins in biological fluids. J. Nanobiotechnol..

[209] Zhang X., Wu Y., Tu Y., Liu S. (2008). A reusable electrochemical immunosensor for carcinoembryonic antigen via molecular recognition of glycoprotein antibody by phenylboronic acid self-assembly layer on gold. Analyst.

[210] Li Y.J., Bi L.J., Zhang X.E., Zhou Y.F., Zhang J.B., Chen Y.Y., Li W., Zhang Z.P. (2006). Reversible immobilization of proteins with streptavidin affinity tags on a surface plasmon resonance biosensor chip. Anal. Bioanal. Chem..

[211] Toner M., Irimia D. (2005). Blood-on-a-chip. Annu. Rev. Biomed. Eng..

[212] Blawas A.S., Reichert W.M. (1998). Protein patterning. Biomaterials.

[213] Kane R.S., Takayama S., Ostuni E., Ingber D.E., Whitesides G.M. (1999). Patterning proteins and cells using soft lithography. Biomater. Silver Jubil. Compend..

[214] Whitesides G.M., Ostuni E., Jiang X., Ingber D.E. (2001). S Oft L Ithography in B Iology. Annu. Rev. Biomed. Eng..

[215] Shirasaki Y., Yamagishi M., Suzuki N., Izawa K., Nakahara A., Mizuno J., Shoji S., Heike T., Harada Y., Nishikomori R. (2014). Real-time single-cell imaging of protein secretion. Sci. Rep..

[216] Love J.C., Ronan J.L., Grotenbreg G.M., Van Der Veen A.G., Ploegh H.L. (2006). A microengraving method for rapid selection of single cells producing antigen-specific antibodies. Nat. Biotechnol..

[217] Seo J.H., Chen L.J., Verkhoturov S.V., Schweikert E.A., Revzin A. (2011). The use of glass substrates with bi-functional silanes for designing micropatterned cell-secreted cytokine immunoassays. Biomaterials.

[218] Yan J., Revzin A., Herold K.E., Rasooly A. (2012). Chapter 22 Micropatterned Biosensing Surfaces for Detection of Cell-Secreted Inflammatory Signals. Biosensors and Molecolar Technology for Cancer Diagnostics.

[219] Zhu H., Stybayeva G., Silangcruz J., Yan J., Ramanculov E., Dandekar S., George M.D., Revzin A. (2009). Detecting cytokine release from single T-cells. Anal. Chem..

[220] Folch A., Toner M. (2000). Microengineering of cellular interactions. Annu. Rev. Biomed. Eng..

[221] Bange A., Halsall H.B., Heineman W.R. (2005). Microfluidic immunosensor systems. Biosens. Bioelectron..

[222] Berthuy O.I., Blum L.J., Marquette C.A. (2016). Cancer-cells on chip for label-free detection of secreted molecules. Biosensors.

[223] Sadik O.A., Aluoch A.O., Zhou A. (2009). Status of biomolecular recognition using electrochemical techniques. Biosens. Bioelectron..

[224] Xu X., Zhang S., Chen H., Kong J. (2009). Integration of electrochemistry in micro-total analysis systems for biochemical assays: Recent developments. Talanta.

[225] Zhang Y.S., Aleman J., Shin S.R., Kilic T., Kim D., Shaegh S.A.M., Massa S., Riahi R., Chae S., Hu N. (2017). Multisensor-integrated organs-on-chips platform for automated and continual in situ monitoring of organoid behaviors. Proc. Natl. Acad. Sci. USA.

[226] Skardal A., Murphy S.V., Devarasetty M., Mead I., Kang H.-W., Seol Y.-J., Zhang Y.S., Shin S.-R., Zhao L., Aleman J. (2017). Multi-tissue interactions in an integrated three-tissue organ-on-a-chip platform. Sci. Rep..

[227] Ortega M.A., Fernández-Garibay X., Castaño A.G., De Chiara F., Hernández-Albors A., Balaguer-Trias J., Ramón-Azcón J. (2019). Muscle-on-a-chip with an on-site multiplexed biosensing system for in situ monitoring of secreted IL-6 and TNF-α. Lab Chip.

[228] Zhu H., Stybayeva G., MacAl M., Ramanculov E., George M.D., Dandekar S., Revzin A. (2008). A microdevice for multiplexed detection of T-cell-secreted cytokines. Lab Chip.

[229] Ma C., Fan R., Ahmad H., Shi Q., Comin-Anduix B., Chodon T., Koya R.C., Liu C.C., Kwong G.A., Radu C.G. (2011). A clinical microchip for evaluation of single immune cells reveals high functional heterogeneity in phenotypically similar T cells. Nat. Med..

[230] Bradshaw E.M., Kent S.C., Tripuraneni V., Orban T., Ploegh H.L., Hafler D.A., Love J.C. (2008). Concurrent detection of secreted products from human lymphocytes by microengraving: Cytokines and antigen-reactive antibodies. Clin. Immunol..

[231] Chattopadhyay P.K., Gierahn T.M., Roederer M., Love J.C. (2014). Single-cell technologies for monitoring immune systems. Nat. Immunol..

[232] Riahi R., Shaegh S.A.M., Ghaderi M., Zhang Y.S., Shin S.R., Aleman J., Massa S., Kim D., Dokmeci M.R., Khademhosseini A. (2016). Automated microfluidic platform of bead-based electrochemical immunosensor integrated with bioreactor for continual monitoring of cell secreted biomarkers. Sci. Rep..

[233] Zhou Q., Kwa T., Gao Y., Liu Y., Rahimian A., Revzin A. (2014). On-chip regeneration of aptasensors for monitoring cell secretion. Lab Chip.

[234] Dishinger J.F., Kennedy R.T. (2007). Serial immunoassays in parallel on a microfluidic chip for monitoring hormone secretion from living cells. Anal. Chem..

[235] Cohen N., Sabhachandani P., Golberg A., Konry T. (2015). Approaching near real-time biosensing: Microfluidic microsphere based biosensor for real-time analyte detection. Biosens. Bioelectron..

[236] Xu Y., Wang E. (2012). Electrochemical biosensors based on magnetic micro/nano particles. Electrochim. Acta.

[237] Son K.J., Gheibi P., Stybayeva G., Rahimian A., Revzin A. (2017). Detecting cell-secreted growth factors in microfluidic devices using bead-based biosensors. Microsystems Nanoeng..

[238] Bini A., Minunni M., Tombelli S., Centi S., Mascini M. (2007). Analytical performances of aptamer-based sensing for thrombin detection. Anal. Chem..

[239] Ferapontova E.E., Olsen E.M., Gothelf K.V. (2008). An RNA aptamer-based electrochemical biosensor for detection of theophylline in serum. J. Am. Chem. Soc..

[240] McCauley T.G., Hamaguchi N., Stanton M. (2003). Aptamer-based biosensor arrays for detection and quantification of biological macromolecules. Anal. Biochem..

[241] Tombelli S., Minunni M., Luzi E., Mascini M. (2005). Aptamer-based biosensors for the detection of HIV-1 Tat protein. Bioelectrochemistry.

[242] Wang J., Jiang Y., Zhou C., Fang X. (2005). Aptamer-based ATP assay using a luminescent light switching complex. Anal. Chem..

[243] Ruigrok V.J.B., Levisson M., Eppink M.H.M., Smidt H., van der Oost J. (2011). Alternative affinity tools: More attractive than antibodies?. Biochem. J..

[244] Maehashi K., Katsura T., Kerman K., Takamura Y., Matsumoto K., Tamiya E. (2007). Label-free protein biosensor based on aptamer-modified carbon nanotube field-effect transistors. Anal. Chem..

[245] Liss M., Petersen B., Wolf H., Prohaska E. (2002). An aptamer-based quartz crystal protein biosensor. Anal. Chem..

[246] Hansen J.A., Wang J., Kawde A.N., Xiang Y., Gothelf K.V., Collins G. (2006). Quantum-dot/aptamer-based ultrasensitive multi-analyte electrochemical biosensor. J. Am. Chem. Soc..

[247] Xiao Y., Lubin A.A., Heeger A.J., Plaxco K.W. (2005). Label-Free Electronic Detection of Thrombin in Blood Serum by Using an Aptamer-Based Sensor. Angew. Chem..

[248] Du L., Wu C., Peng H., Zou L., Zhao L., Huang L., Wang P. (2013). Piezoelectric olfactory receptor biosensor prepared by aptamer-assisted immobilization. Sens. Actuators B Chem..

[249] Liu Y., Tuleouva N., Ramanculov E., Revzin A. (2010). Aptamer-based electrochemical biosensor for interferon gamma detection. Anal. Chem..

[250] Kara P., de la Escosura-Muñiz A., Maltez-da Costa M., Guix M., Ozsoz M., Merkoçi A. (2010). Aptamers based electrochemical biosensor for protein detection using carbon nanotubes platforms. Biosens. Bioelectron..

[251] Jayasena S.D. (1999). Aptamers: An emerging class of molecules that rival antibodies in diagnostics. Clin. Chem..

[252] Sun H., Zhu X., Lu P.Y., Rosato R.R., Tan W., Zu Y. (2014). Oligonucleotide aptamers: New tools for targeted cancer therapy. Mol. Ther. Nucleic Acids.

[253] Shaban S.M., Kim D.-H. (2021). Recent Advances in Aptamer Sensors. Sensors.

[254] Li J.J., Fang X., Tan W. (2002). Molecular aptamer beacons for real-time protein recognition. Biochem. Biophys. Res. Commun..

[255] Xiao Y., Piorek B.D., Plaxco K.W., Heegert A.J. (2005). A reagentless signal-on architecture for electronic, aptamer-based sensors via target-induced strand displacement. J. Am. Chem. Soc..

[256] Radi A.E., Acero Sánchez J.L., Baldrich E., O’Sullivan C.K. (2006). Reagentless, reusable, ultrasensitive electrochemical molecular beacon aptasensor. J. Am. Chem. Soc..

[257] Nguyen T.H., Hilton J.P., Lin Q. (2009). Emerging applications of aptamers to micro- and nanoscale biosensing. Microfluid. Nanofluidics.

[258] Liu Y., Yan J., Howland M.C., Kwa T., Revzin A. (2011). Micropatterned aptasensors for continuous monitoring of cytokine release from human leukocytes. Anal. Chem..

[259] Liu Y., Kwa T., Revzin A. (2012). Simultaneous detection of cell-secreted TNF-α and IFN-γ using micropatterned aptamer-modified electrodes. Biomaterials.

[260] Liu Y., Liu Y., Matharu Z., Rahimian A., Revzin A. (2015). Detecting multiple cell-secreted cytokines from the same aptamer-functionalized electrode. Biosens. Bioelectron..

[261] Kwa T., Zhou Q., Gao Y., Rahimian A., Kwon L., Liu Y., Revzin A. (2014). Reconfigurable microfluidics with integrated aptasensors for monitoring intercellular communication. Lab Chip.

[262] Shin S.R., Zhang Y.S., Kim D.J., Manbohi A., Avci H., Silvestri A., Aleman J., Hu N., Kilic T., Keung W. (2016). Aptamer-Based Microfluidic Electrochemical Biosensor for Monitoring Cell-Secreted Trace Cardiac Biomarkers. Anal. Chem..

[263] Lee J., Mehrotra S., Zare-Eelanjegh E., Rodrigues R.O., Akbarinejad A., Ge D., Amato L., Kiaee K., Fang Y.C., Rosenkranz A. (2020). A Heart-Breast Cancer-on-a-Chip Platform for Disease Modeling and Monitoring of Cardiotoxicity Induced by Cancer Chemotherapy. Small.

[264] Shin Y., Han S., Jeon J.S., Yamamoto K., Zervantonakis I.K., Sudo R., Kamm R.D., Chung S. (2012). Microfluidic assay for simultaneous culture of multiple cell types on surfaces or within hydrogels. Nat. Protoc..

[265] Marrella A., Buratti P., Markus J., Firpo G., Pesenti M., Landry T., Ayehunie S., Scaglione S., Kandarova H., Aiello M. (2020). In vitro demonstration of intestinal absorption mechanisms of different sugars using 3d organotypic tissues in a fluidic device. ALTEX.

[266] Schoukroun-Barnes L.R., Wagan S., Liu J., Leach J.B., White R.J. (2013). Biocompatible hydrogel membranes for the protection of RNA aptamer-based electrochemical sensors. Smart Biomed. Physiol. Sens. Technol. X.

[267] Santos-Cancel M., White R.J. (2017). Collagen Membranes with Ribonuclease Inhibitors for Long-Term Stability of Electrochemical Aptamer-Based Sensors Employing RNA. Anal. Chem..

[268] Rowe A.A., Miller E.A., Plaxco K.W. (2010). Reagentless measurement of aminoglycoside antibiotics in blood serum via an electrochemical, ribonucleic acid aptamer-based biosensor. Anal. Chem..

[269] Ferapontova E.E., Gothelf K.V. (2009). Optimization of the electrochemical RNA-aptamer based biosensor for theophylline by using a methylene blue redox label. Electroanalysis.

[270] Verderio C., Matteoli M. (2011). ATP in neuron-glia bidirectional signalling. Brain Res. Rev..

[271] Savtchouk I., Volterra A. (2018). Gliotransmission: Beyond black-and-white. J. Neurosci..

[272] Santos-Cancel M., Simpson L.W., Leach J.B., White R.J. (2019). Direct, Real-Time Detection of Adenosine Triphosphate Release from Astrocytes in Three-Dimensional Culture Using an Integrated Electrochemical Aptamer-Based Sensor. ACS Chem. Neurosci..

